# TRAIL receptor agonists convert the response of breast cancer cells to ONC201 from anti-proliferative to apoptotic

**DOI:** 10.18632/oncotarget.27773

**Published:** 2020-10-20

**Authors:** Marie D. Ralff, Aakash Jhaveri, Jocelyn E. Ray, Lanlan Zhou, Avital Lev, Kerry S. Campbell, David T. Dicker, Eric A. Ross, Wafik S. El-Deiry

**Affiliations:** ^1^MD/PhD Program, The Lewis Katz School of Medicine, Temple University, Philadelphia, PA, USA; ^2^Laboratory of Translational Oncology and Experimental Cancer Therapeutics, Department of Medical Oncology and Molecular Therapeutics Program, Fox Chase Cancer Center, Philadelphia, PA, USA; ^3^Master of Science in Biotechnology Program, The Warren Alpert Medical School, Brown University, Providence, RI, USA; ^4^Joint Program in Cancer Biology, Brown University and the Lifespan Health System, Providence, RI, USA; ^5^Laboratory of Translational Oncology and Experimental Cancer Therapeutics, Department of Pathology and Laboratory Medicine, The Warren Alpert Medical School, Brown University, Providence, RI, USA; ^6^Division of Gynecologic Oncology, Fox Chase Cancer Center, Philadelphia, PA, USA; ^7^Blood Cell Development and Function Program, Fox Chase Cancer Center, Philadelphia, PA, USA; ^8^Biostatistics and Bioinformatics Facility, Fox Chase Cancer Center, Philadelphia, PA, USA; ^9^Hematology-Oncology Division, Brown University and the Lifespan Cancer Institute, Providence, RI, USA; ^10^Cancer Center at Brown University, The Warren Alpert Medical School, Brown University, Providence, RI, USA

**Keywords:** ONC201, TRAIL, breast cancer, death receptors, apoptosis

## Abstract

ONC201 was initially identified as an inducer of cell death through the tumor necrosis factor-related apoptosis-inducing ligand (TRAIL) pathway. The compound is currently being tested in patients with hematological malignancies and solid tumors, including those of the breast. We investigated strategies to convert the response of breast cancers to ONC201 from anti-proliferative to apoptotic. ONC201 treatment upregulates TRAIL and primes TRAIL-resistant non-triple negative breast cancer (TNBC) cells to undergo cell death through the extrinsic pathway. Remarkably, the addition of exogenous recombinant human TRAIL (rhTRAIL) converts the response of TRAIL-resistant non-TNBC cells to ONC201 from anti-proliferative to apoptotic in a death receptor 5 (DR5)-dependent manner *in vitro*. Importantly, normal fibroblasts do not undergo apoptosis following rhTRAIL plus ONC201. *In vivo*, MDA-MB-361 tumor growth rate is significantly reduced following treatment with a combination of ONC201 and rhTRAIL as compared to control tumors. Natural killer (NK) cells which use TRAIL to kill DR5-expressing cancer cells, exhibit greater cytotoxicity against ONC201-treated breast cancer cells compared to controls. rhTRAIL also converts the response of cells from other tumor types to ONC201 from anti-proliferative to apoptotic. A monoclonal DR5-agonistic antibody converts the response of non-TNBC cells to ONC201 from anti-proliferative to apoptotic. Our findings describe a novel therapeutic strategy that potently converts the response of a cancer cell to ONC201 from anti-proliferative to apoptotic. This approach may be clinically relevant and has potential to induce tumor regression of patient tumors with relative resistance to ONC201 monotherapy.

## INTRODUCTION

Breast cancer is the most commonly diagnosed cancer and is the number three cause of cancer-related death in United States women. There is an urgent need for the development of novel breast cancer therapeutics with increased efficacy and decreased toxicity. One promising therapeutic is ONC201, a compound being tested clinically in recurrent or refractory metastatic breast cancer (NCT03394027). ONC201 was initially identified as a tumor necrosis factor-related apoptosis-inducing ligand (TRAIL) pathway inducer [1]. TRAIL is an activator of apoptosis through the extrinsic pathway [2, 3] that selectively targets transformed cells [4] through binding to cell surface receptors death receptor 4 (DR4) [5] and death receptor 5 (DR5) [6, 7]. We previously identified DR5 as a p53-regulated death receptor [6]. The potential of TRAIL to kill cancerous cells while leaving normal cells unharmed led to the development and clinical testing of TRAIL-based therapies such as recombinant human TRAIL (rhTRAIL; APO2L/TRAIL; dulanermin) and death receptor agonistic antibodies. Ultimately, these TRAIL-based therapies showed limited clinical efficacy, leading to investigation of alternative strategies to activate this potent pro-apoptotic pathway.

Our lab discovered ONC201, the founding member of a class of compounds known as the imipridones, in a screen for small molecules capable of transcriptionally inducing the TRAIL gene. ONC201 (originally TIC10) potently induced cell death through the extrinsic pathway in cancer cells from a variety of tumor types [1]. The compound has since completed early phase clinical testing in advanced solid tumors where it was shown to be safe and to have a preliminary efficacy signal [8, 9]. ONC201 is currently being tested in phase II clinical trials in patients with a variety of solid tumors and hematological malignancies [10]. The compound is unique in that it is a dual activator of the TRAIL pathway, able not only to upregulate pro-death ligand TRAIL, and but also its receptor DR5. Early studies of the mechanism of action of ONC201 showed that the compound inhibited pro-survival kinases Akt and ERK, leading to the dephosphorylation and activation of transcription factor FOXO3a. Active FOXO3a translocated into the nucleus, where it induced transcription of the TRAIL gene [1]. Later work described the ability of the compound to upregulate the TRAIL receptor DR5 in an ATF4- and CHOP-dependent manner as part of an integrated stress response (ISR) [11].

Most breast cancers are highly resistant to TRAIL, with the exception of a subset of triple negative breast cancers (TNBC) [12]. A number of resistance mechanisms to TRAIL in breast cancer have been identified. At the cell surface, constitutive endocytosis of death receptors can contribute to TRAIL resistance [13]. Intracellularly, cellular FLICE inhibitory protein (cFLIP) can compete with caspase-8 for binding to death receptor-associated FADD, preventing proper formation of the death inducing signaling complex (DISC) [14]. In type II cells, high levels of anti-apoptotic Bcl-2 family proteins Bcl-2, Mcl-1, and Bcl-XL can contribute to TRAIL resistance [15]. Further downstream, inhibitor of apoptosis (IAP) family proteins such as XIAP and c-IAP1 can directly or indirectly inhibit the activity of effector caspases 3 and 7 [16, 17] and thus modulate sensitivity to TRAIL.

Our lab showed that ONC201 treatment decreased the viability of cancer cells from a wide variety of tumor types irrespective of whether or not it induced apoptosis [11]. An inhibition of cellular proliferation was characterized by decreased cyclin D1 protein levels and an arrest in the G_1_ phase of the cell cycle [11]. Given our knowledge that ONC201 could inhibit proliferation in addition to inducing TRAIL-dependent apoptosis, we tested the efficacy of ONC201 in a panel of TRAIL-sensitive and TRAIL-resistant TNBC and non-TNBC cells. ONC201 showed sweeping efficacy across the panel, decreasing cell viability irrespective of the molecular subtype or TRAIL-sensitivity of the cells [18].

Further characterization of the response of breast cancer cells to ONC201 revealed that the anti-proliferative effect was more common than the apoptotic effect. The apoptotic effect of the compound was TRAIL-dependent and led to *in vivo* efficacy in a xenograft model [18], similar to what had been observed in other tumor types [1]. Intriguingly, the more common anti-proliferative effect involving a G_1_ arrest did not lead to *in vivo* efficacy of the compound [18].

The goal of the present study was to investigate underlying mechanisms related to this issue and to identify novel therapeutic strategies for converting the effects of ONC201 in breast cancer from anti-proliferative to apoptotic. Previous work has shown that ONC201 is a potent dual inducer of the TRAIL pathway at the level of both the ligand [1] and the receptor [11], and that breast cancers show decreased sensitivity to TRAIL [12]. We hypothesized that profiling the effects of the compound on the TRAIL pathway in breast cancer and identifying blocks in signal transduction would allow us to identify therapeutic strategies with the potential to induce apoptosis and that could potentially translate to tumor regressions in patients who do not respond to treatment with ONC201 alone.

## RESULTS

### ONC201 has anti-proliferative effects against TRAIL-resistant non-TNBC cells

**Figure 1:**
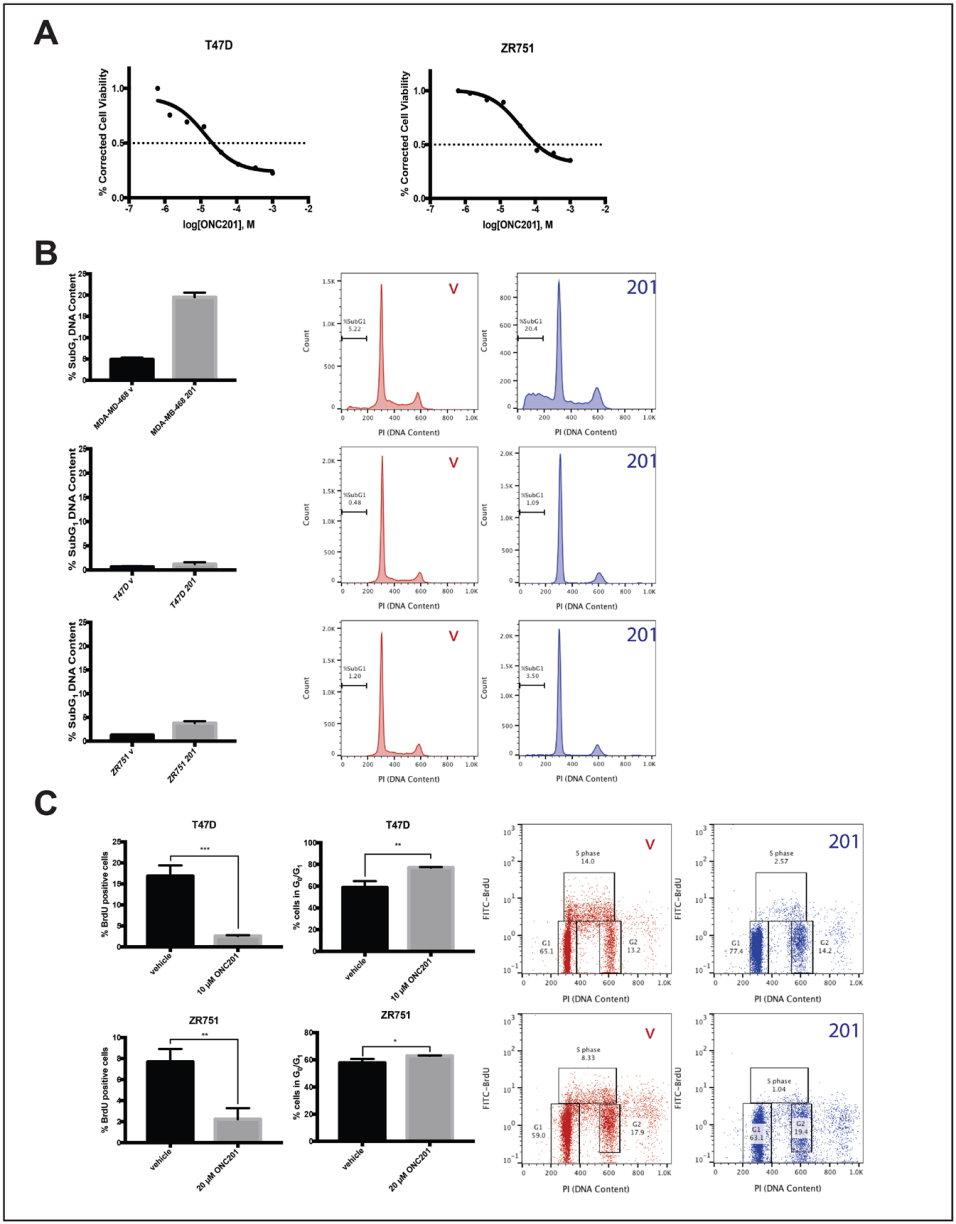
ONC201 inhibits the proliferation but does not induce apoptosis in T47D and ZR751 TRAIL-resistant non-TNBC cells. (**A**) Dose response curves for cells treated with varying concentrations of ONC201 for 72 hours were generated. Cell viability was determined using CellTiterGlo reagent. (**B**) Cells were treated with a vehicle control or approximate GI_50_ doses of ONC201 for 72 hours and stained with propidium iodide. Flow cytometric analysis of the cells was used to determine the percentage of cells with subG_1_ DNA content. (**C**) Cells were treated with a vehicle control or approximate GI_50_ doses of ONC201 for 48 hours, then pulsed with BrdU for 30 minutes. BrdU-PI staining was performed and the % BrdU positive cells quantitated using flow cytometric analysis. Representative dot plots are shown. Experiments shown in this figure were conducted in triplicate. ns: *p* > 0.05; ^*^ *p* ≤ 0.05, ^**^ *p* ≤ 0.01, ^***^ *p* ≤ 0.001, ^****^ *p* ≤ 0.0001.

We previously showed that most breast cancer cells lines were resistant to TRAIL and did not undergo cell death following treatment with ONC201 [18]. Here we select four of these cell lines and further characterize their response to ONC201. We use low micromolar doses of the ONC201 compound shown to be clinically achievable in the first-in-human trial [9]. Treatment of TRAIL-resistant non-TNBC cells with ONC201 for 72 hours leads to a clear decrease in cell viability (Figure 1A, Supplementary Figure 1A). However, cell cycle profiling following propidium iodide staining indicates that the percent of non-TNBC cells with subG_1_ DNA content does not increase from that observed in the vehicle control (Figure 1B, Supplementary Figure 1B). This is in contrast to the four-fold increase in MDA-MB-468 (Figure 1B), a cell line with known sensitivity to the TRAIL-dependent apoptotic effects of ONC201 [18]. Flow cytometric BrdU-PI staining indicates that uptake of nucleoside analog BrdU into the DNA decreases in a statistically significant manner following a 48-hour treatment with ONC201 in all non-TNBC cell lines tested (Figure 1C, Supplementary Figure 1C). This is accompanied by an increase in the percent of cells with a G_0_/G_1_ DNA content, indicative of an arrest in the G_1_ phase of the cell cycle. (Figure 1C, Supplementary Figure 1C). Together, these results show that the effects of ONC201 in TRAIL-resistant non-TNBC cells are anti-proliferative rather than apoptotic and involve an arrest in the G_1_ phase of the cell cycle.

### ONC201-treated TRAIL-resistant non-TNBC cells upregulate TRAIL and are primed to undergo TRAIL-dependent cell death

**Figure 2:**
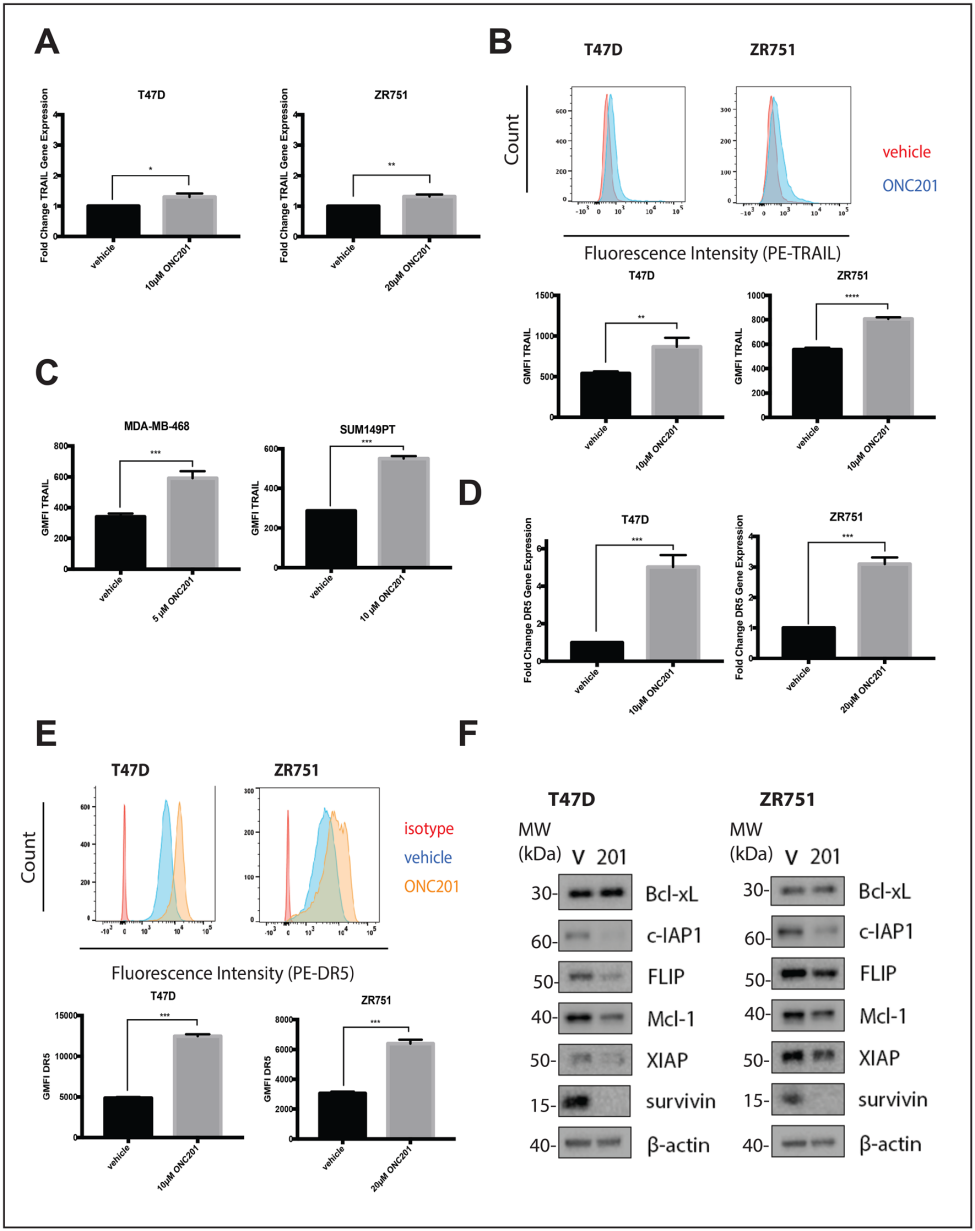
ONC201 treated T47D and ZR751 non-TNBC cells increase TRAIL expression and are primed to undergo TRAIL-dependent apoptosis. (**A**) Cells were treated with a vehicle control or approximate GI_50_ doses of ONC201 for 48 hours and qRT-PCR was used to determine the fold change in TRAIL gene expression. (**B**) Cells were treated with a vehicle control or approximate GI_50_ doses of ONC201 for 72 hours and then stained with an anti-TRAIL antibody. The geometric mean fluorescence intensity was determined using flow cytometric analysis. Representative histograms show the staining. (**C**) Cells were treated with a vehicle control or approximate GI_50_ doses of ONC201 for 72 hours and then stained with an anti-TRAIL antibody. The geometric mean fluorescence intensity was determined using flow cytometric analysis. (**D**) Cells were treated with a vehicle control or approximate GI_50_ doses of ONC201 for 48 hours and qRT-PCR was used to determine the fold change in DR5 gene expression. (**E**) Cells were treated with a vehicle control or approximate GI_50_ doses of ONC201 for 72 hours and then stained with an isotype control or an anti-DR5 antibody. The geometric mean fluorescence intensity was determined using flow cytometric analysis. Representative histograms show the staining. (**F**) Cells were treated with a vehicle control or GI_50_ doses of ONC201 for 72 hours. Western blot analysis used to assess expression of anti-apoptotic proteins. Experiments shown in this figure were conducted in triplicate. ns: *p* > 0.05; ^*^ *p* ≤ 0.05, ^**^ *p* ≤ 0.01, ^***^ *p* ≤ 0.001, ^****^ *p* ≤ 0.0001.

ONC201 is a TRAIL pathway inducer and the pro-apoptotic effects of the compound have been previously shown to be TRAIL-dependent [1]. We hypothesized that in non-TNBC cells, resistance to the TRAIL produced following ONC201 treatment was responsible for the fact that the effects of the compound were anti-proliferative but not apoptotic. We first confirmed that ONC201 treatment in non-TNBC cells leads to the upregulation of TRAIL. Treatment of breast cancer cells with ONC201 or a vehicle control for 48 hours leads to small but statistically significant increases in TRAIL mRNA levels in the ONC201 treated cells (Figure 2A, Supplementary Figure 2A). In addition, we observe small but statistically significant increases in surface TRAIL protein in cells treated with ONC201 for 72 hours when compared to vehicle treated cells (Figure 2B, Supplementary Figure 2B). Although the increases in TRAIL in these cells are minimal, surface TRAIL induction following ONC201 treatment in breast cancer cells where the effects of the compound involve TRAIL-dependent apoptosis (MDA-MB-468, SUM149PT) [18] is also less than 2-fold above vehicle (Figure 2C).

TRAIL resistance occurs when signal transduction through the pathway is blocked at one or more points. In cells sensitive to the apoptotic effects of ONC201, treatment with the compound upregulated surface levels of TRAIL receptor DR5 [1], the main death receptor used by breast cancer cells [19]. We hypothesized that a lack of DR5 induction might explain why the effects of ONC201 in non-TNBC cells were anti-proliferative but not apoptotic. However, we observe a statistically significant increase in DR5 mRNA levels in breast cancer cells treated with ONC201 for 48 hours when compared with a vehicle control (Figure 2D, Supplementary Figure 2C). In addition, significantly more DR5 protein is present on the surface of the cells treated for 72 hours with ONC201 compared to those treated with a vehicle control (Figure 2E, Supplementary Figure 2D).

We investigated whether high levels of anti-apoptotic proteins including cFLIP and those in the Bcl-2 and IAP families might be responsible for the lack of apoptosis induction following ONC201 treatment. We observe that a 72-hour treatment of breast cancer cells with ONC201 leads to decreased expression of some or all of these anti-apoptotic proteins (Figure 2F, Supplementary Figure 2E). Changes in RNA do not correlate with what is observed with these anti-apoptotic proteins at the protein level (Supplementary Figure 3). Together, our findings indicate that TRAIL-resistant non-TNBC cells upregulate TRAIL and are primed to undergo TRAIL-dependent cell death following treatment with ONC201, expressing high levels of death receptors and low levels of anti-apoptotic proteins.

### The response of breast cancer cells to ONC201 is converted from anti-proliferative to pro-apoptotic by rhTRAIL in a DR5-dependent manner

**Figure 3:**
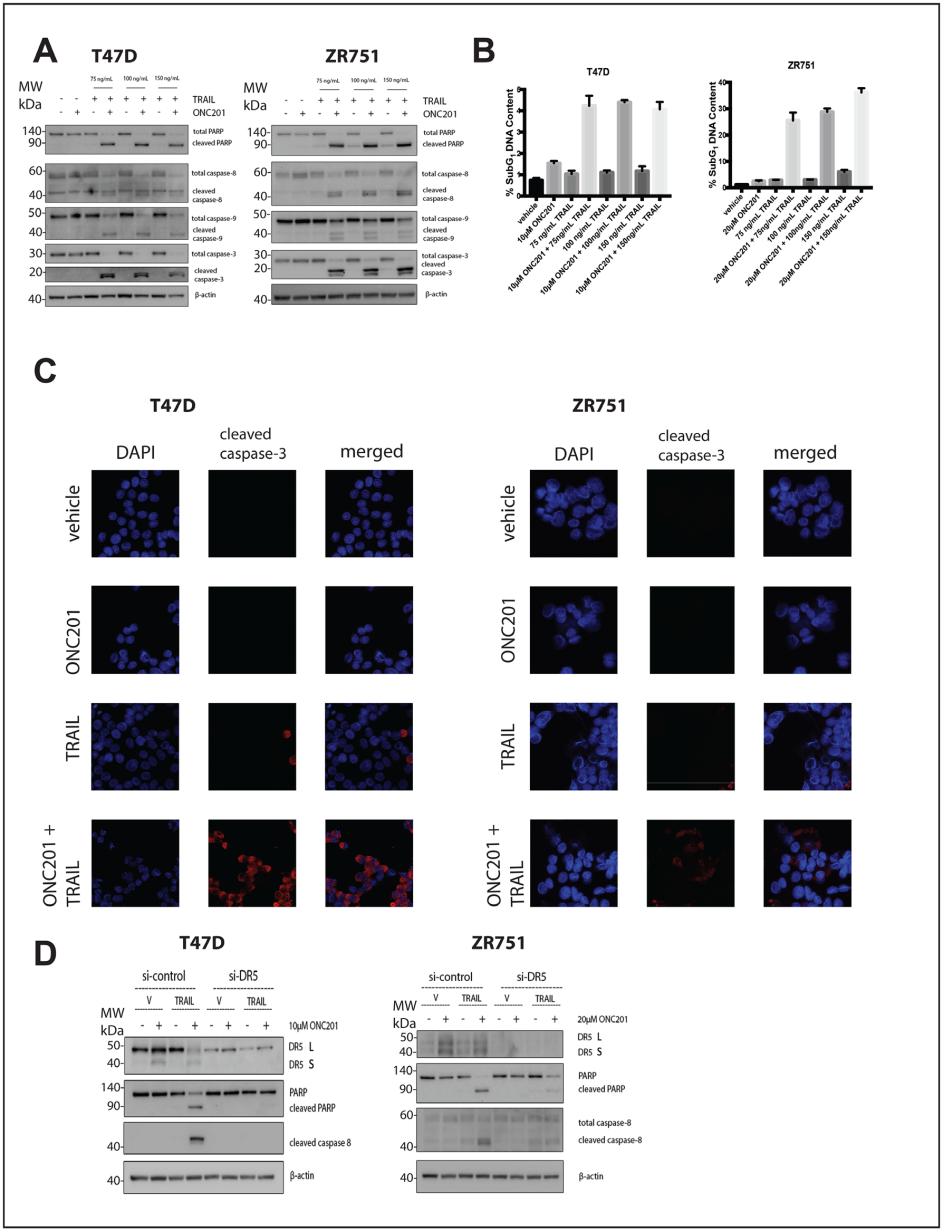
Addition of rhTRAIL converts the response of T47D and ZR751 breast cancer cells to ONC201 from anti-proliferative to pro-apoptotic in a DR5-dependent manner. (**A**) T47D and ZR751 cells were treated with a vehicle control or GI_50_ doses of ONC201 for 72 hours. Recombinant human TRAIL (rhTRAIL) was added for 4 hours and the induction of cell death was assessed using western blot. (**B**) T47D and ZR751 cells were treated as above and stained with propidium iodide. Flow cytometric analysis of the cells was used to determine the percentage of cells with subG_1_ DNA content. (**C**) T47D cells were treated with a vehicle control or GI_50_ doses of ONC201 for 72 hours. Recombinant human TRAIL (rhTRAIL) was added for 4 hours and cells were fixed and stained for cleaved caspase-3. (**D**) T47D and ZR751 cells were transfected with a scrambled siRNA or a DR5-targeting siRNA for 18 hours, then treated with a vehicle control or GI_50_ doses of ONC201 for 48 hours. Recombinant human TRAIL (rhTRAIL) at 100 ng/mL was added for 4 hours and western blot analysis used to assess induction of cell death. Experiments shown in this figure were conducted in triplicate.

We investigated whether the lack of cell death following ONC201 treatment could be explained by relatively low levels of TRAIL induction in TRAIL-resistant non-TNBC cells. Treatment of breast cancer cells with a vehicle control, ONC201 alone, or recombinant human TRAIL (rhTRAIL) alone does not induce PARP or caspase cleavage (Figure 3A, Supplementary Figure 4A). However, pretreatment with ONC201 for 72 hours followed by a 4-hour incubation with rhTRAIL at three different doses leads to potent caspase and PARP cleavage (Figure 3A, Supplementary Figure 4A). Cell cycle profiling following propidium iodide staining further confirms this result, as cells treated with ONC201 followed by rhTRAIL show a significantly larger population with subG_1_ DNA content as compared to cells treated with a vehicle control, ONC201 alone, or rhTRAIL alone (Figure 3B, Supplementary Figure 4B). Immunofluorescence analysis indicated that cells treated with ONC201 for 72 hours followed by rhTRAIL for 4 hours stain positively for cleaved caspase-3 whereas those treated with a vehicle control, ONC201 alone, or rhTRAIL alone do not (Figure 3C, Supplementary Figure 4C). Our findings are relevant in other tumor types as well, increasing their translational potential, as the addition of rhTRAIL to ONC201-treated lung, glioblastoma, colon, and pancreatic cancer cells also converts their response to the compound from anti-proliferative to apoptotic (Figure 6).

**Figure 4:**
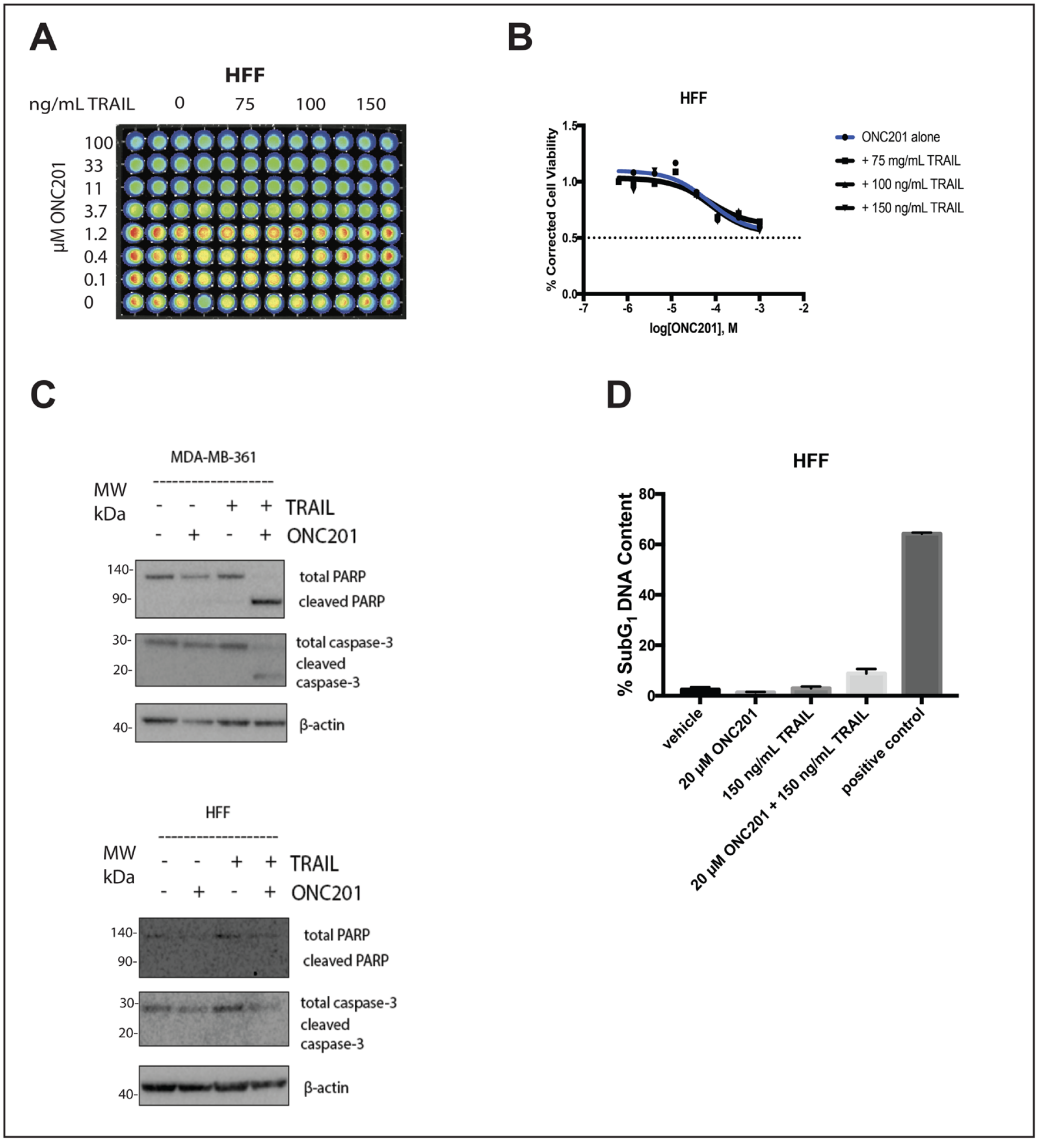
Addition of rhTRAIL does not convert the response to the compound in normal fibroblasts from anti-proliferative to apoptotic. (**A**) HFF cells were treated with a vehicle control or increasing concentrations of ONC201 for 72 hours. rhTRAIL was added for 4 hours and cell viability determined using CellTiterGlo reagent. Luminescent images of the 96 well plates are shown. (**B**) Dose-response curves generated from the quantitation of the data in (A). (**C**) HFF and MDA-MB-361 cells were treated as above and the induction of cell death was assessed using western blot. (**D**) HFF and ZR751 cells were treated as above and stained with propidium iodide. Flow cytometric analysis of the cells was used to determine the percentage of cells with subG_1_ DNA content. Experiments shown in this figure were conducted in triplicate.

**Figure 5:**
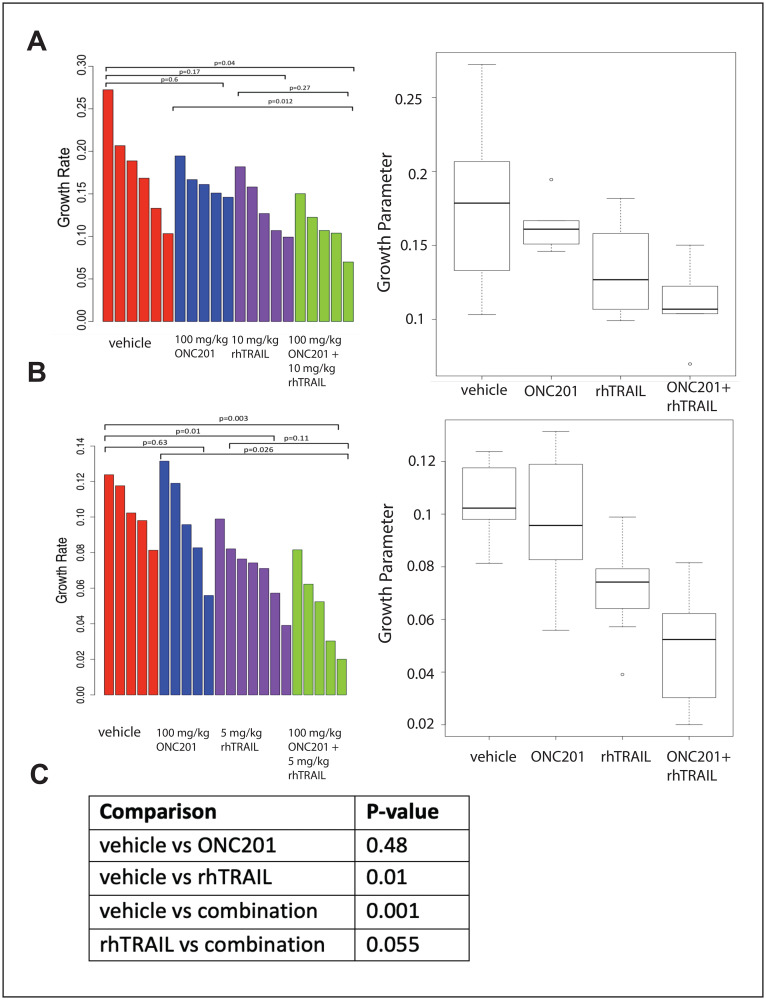
Combination of rhTRAIL and ONC201 in the MDA-MB-361 xenograft model. (**A**) Growth rates were calculated for MDA-MB-361 xenograft tumors were treated weekly with a vehicle control, 100 mg/kg ONC201 orally, 10 mg/kg rhTRAIL intravenously, or a combination of ONC201 and rhTRAIL. rhTRAIL was given 72 hours after ONC201. Mice were treated for a total of three cycles. (**B**) Mice bearing MDA-MB-361 xenograft tumors were treated weekly with a vehicle control, 100 mg/kg ONC201 orally, 5 mg/kg rhTRAIL intravenously, or a combination of ONC201 and rhTRAIL. rhTRAIL was given 72 hours after ONC201. Mice were treated for a total of three cycles. (**C**) Data sets in A and B were combined and re-analyzed. Tumor volumes were measured as (w^2^ × l)/2 where w is tumor width and l is tumor length. 5–7 tumors were included per group.

**Figure 6:**
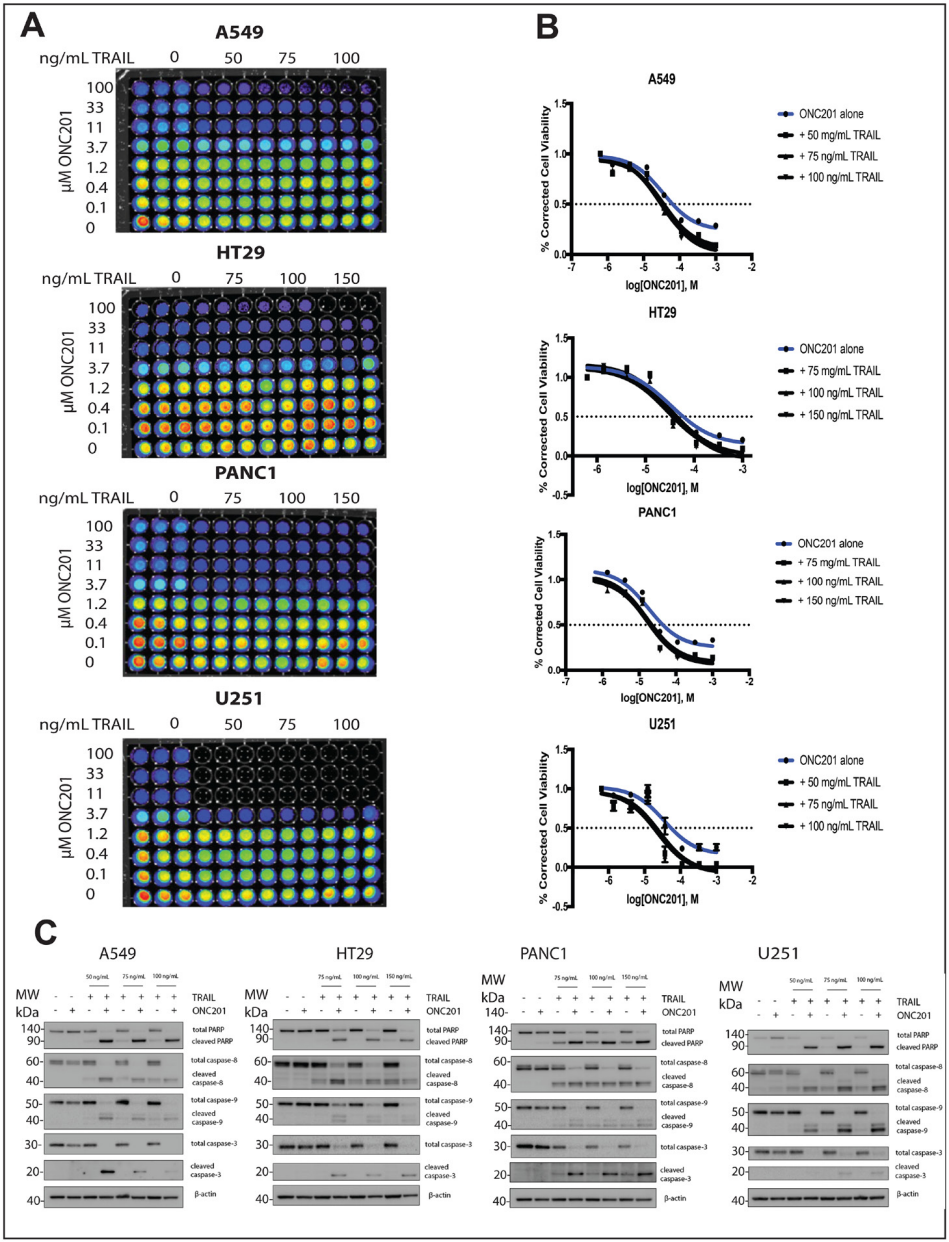
Addition of rhTRAIL converts the response of brain, pancreatic, lung, and colon cancer cells to ONC201 from anti-proliferative to pro-apoptotic. (**A**) A549, HT29, PANC1, and U251 were treated with a vehicle control or increasing concentrations of ONC201 for 72 hours. rhTRAIL was added for 4 hours and cell viability determined using CellTiterGlo reagent. Luminescent images of the 96 well plates are shown. (**B**) Dose-response curves generated from the quantitation of the data in (A). (**C**) A549, HT29, PANC1, and U251 cells were treated as above and the induction of cell death was assessed using western blot. Experiments shown in this figure were conducted in triplicate.

We investigated whether DR5 upregulation by ONC201 was required for rhTRAIL to convert the response to the compound from anti-proliferative to pro-apoptotic in breast cancer. Breast cancer cells were transfected with scrambled siRNA or DR5-targeting siRNA. The mature form of DR5 is clearly upregulated following 48-hour ONC201 treatment in the cells transfected with the scrambled siRNA but not the DR5-targeting siRNA. The apoptosis observed following sequential treatment with ONC201 and rhTRAIL is blunted or abrogated in the si-DR5 transfected cells when compared with the scrambled siRNA transfected cells (Figure 3D, Supplementary Figure 4D). Together, these results show that rhTRAIL converts the response of breast cancer cells to ONC201 from anti-proliferative to pro-apoptotic in a manner that is dependent upon DR5 upregulation by the compound.

### In normal fibroblasts, rhTRAIL does not convert the response to ONC201 from anti-proliferative to apoptotic

Importantly, the addition of rhTRAIL to ONC201-treated normal fibroblast cells does not convert their response to the compound from anti-proliferative to apoptotic (Figure 4). ONC201 treatment alone for 72 hours slightly decreases the viability of HFF cells at high micromolar doses, but the addition of rhTRAIL at doses comparable to those used in cancer cells for 4 hours does not decrease the viability further (Figure 4A and 4B). Western blot analysis indicates that while the combination of rhTRAIL and ONC201 induces PARP and caspase-3 cleavage in MDA-MB-361 breast cancer cells, it does not have this effect in HFF normal fibroblast cells (Figure 4C). This result is further confirmed by propidium iodide staining and cell cycle profiling, as the combination of rhTRAIL and ONC201 induces cell death in ZR751 but not in HFF cells (Figure 4D).

### The combination of ONC201 and rhTRAIL *in vivo* reduces the growth rate of MDA-MB-361 xenograft tumors

There is a statistically significant difference in the growth rate of MDA-MB-361 tumors from mice treated with a combination of 100 mg/kg ONC201 orally and 10 mg/kg rhTRAIL IV when compared with tumors from mice treated with a vehicle control. (Figure 5A). A similar trend is noted when comparing MDA-MB-361 tumors from mice treated with a combination of 100 mg/kg ONC201 orally and 5 mg/kg rhTRAIL IV when compared with mice treated with a vehicle control. There is a trend towards significance in the difference in the growth rates of tumors treated with rhTRAIL IV alone and tumors treated with a combination of 100 mg/kg ONC201 orally and rhTRAIL IV (*p* = 0.27, Figure 5A; *p* = 0.11, Figure 5B). When the data sets are combined, there is an even stronger trend towards significance in the difference in the growth rates of tumors from mice treated with rhTRAIL IV alone and the growth rates of tumors from mice treated with a combination of ONC201 orally and 10 mg/kg rhTRAIL IV (*p* = 0.055) (Figure 5C).

The weights of mice treated with a vehicle control, 100 mg/kg ONC201, 10 mg/kg rhTRAIL, or the combination of ONC201 and rhTRAIL remain stable over three treatment cycles (Supplementary Figure 6A). The livers from mice treated with a combination of rhTRAIL and ONC201 appear histologically normal, similar to the livers of mice treated with a vehicle control, 100 mg/kg ONC201 alone, and 5 mg/kg rhTRAIL alone (Supplementary Figure 6B).

Preliminary biomarker studies in MDA-MB-361 xenografts treated with ONC201, rhTRAIL, and the combination did not show decreased Ki67 staining intensity or decreased cleaved caspase-3 positive cell count (data not shown). However, the percentage of high intensity Ki-67 positive nuclei is less in in MDA-MB-436 and MDA-MB-231 tumors treated with a combination of rhTRAIL and ONC201 when compared with a vehicle control, 100 mg/kg ONC201 alone, or 5 mg/kg rhTRAIL alone (Supplementary Figures 7 and 8).

### NK cells exhibit greater cytotoxicity against ONC201-treated tumor cells in comparison to vehicle treated cells

Our lab recently showed that in addition to its effects on apoptosis and cellular proliferation, ONC201 treatment led to the recruitment of natural killer (NK) cells to tumors *in vivo* [20]. NK cells are TRAIL-producing lymphoid cells with an important role in controlling tumor progression [21, 22]. We hypothesized that a similar recruitment of NK cells would be observed in ONC201 treated mouse breast tumors. Indeed, C57BL/6 mice bearing E0771 tumors in the mammary fat pad and treated with ONC201 showed a significantly increased percentage of NK cells (NK1.1^+^, CD3^-^, CD19^-^) within the tumor infiltrating lymphocytes (CD45^+^) (Supplementary Figure 5A). TRAIL is expressed on the surface of activated NK cells [23] and can induce apoptosis in tumor cells through binding to death receptors on the cell surface [24, 25]. ONC201 treatment leads to the upregulation of TRAIL receptor DR5 in breast cancer cells (Figure 2A and 2B). We hypothesized that breast cancer cells pre-treated with ONC201 would be more sensitive to the cytotoxic effects of NK cells. We performed a cell-based cytotoxicity assay involving the co-culture of T47D and NK92 cells. NK92 cells killed T47D breast cancer cells with a greater efficiency when the T47D cells had been pre-treated with ONC201 at both a 2:1 and 4:1 effector to target (E: T) ratio (Supplementary Figure 5B). These data indicate that NK cells are recruited to mouse breast tumors following ONC201 treatment, and that NK cells kill breast cancer cells *in vitro* with greater efficacy when they have been pre-treated with ONC201.

### The response of breast cancer cells can be converted from anti-proliferative to pro-apoptotic by a DR5-agonistic antibody

**Figure 7:**
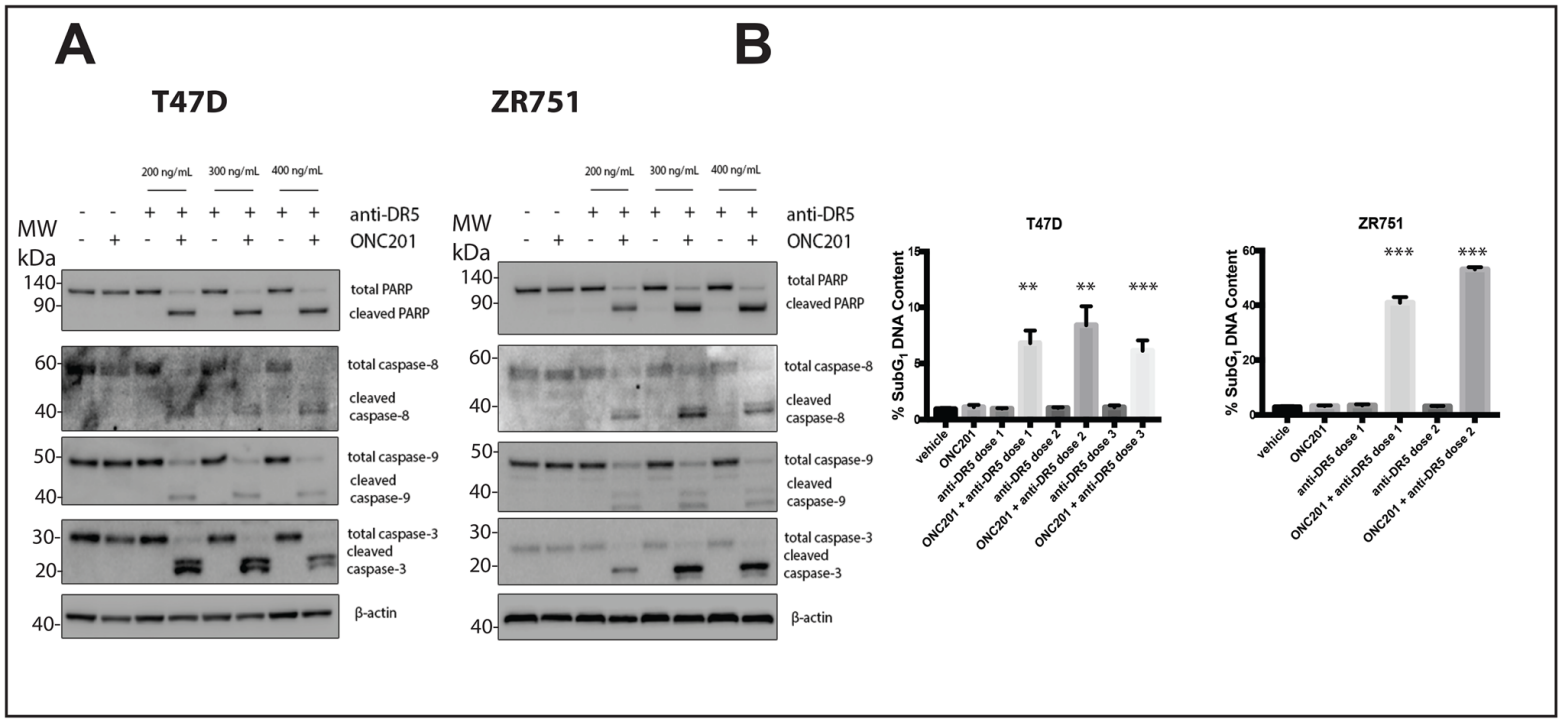
A DR5-agonistic antibody can also be used to convert the response of T47D and ZR751 breast cancer cells from anti-proliferative to pro-apoptotic. (**A**) T47D and ZR751 cells were treated with a vehicle control or ONC201 for 72 hours. Anti-DR5 antibody was added at varying concentrations for four hours and the induction of cell death was assessed using western blot. (**B**) T47D and ZR751 cells were treated as above and stained with propidium iodide. Specifically, ZR751 cells were treated with 200 and 300 ng/mL anti-DR5 antibody. Flow cytometric analysis of the cells was used to determine the percentage of cells with subG_1_ DNA content. Experiments shown in this figure were conducted in triplicate.

DR5-agonistic antibodies are another form of TRAIL receptor agonist (TRA) that has been widely tested in clinical trials [15]. TRAIL-resistant non-TNBC cells are also resistant to a DR5-agonistic antibody, while TRAIL-sensitive TNBC cell line MDA-MB-231 is not (Supplementary Figure 9A). Treatment of breast cancer cells with vehicle, ONC201, or DR5-agonistic antibody alone does not lead to caspase or PARP cleavage (Figure 7A, Supplementary Figure 9B). However, when cells were pre-treated with ONC201 for 72 hours following treatment with a DR5-agonistic antibody for four hours, PARP and the caspases are cleaved, indicating induction of apoptosis (Figure 7A, Supplementary Figure 9B). Similar results are observed following propidium iodide staining, where treatment with ONC201 for 72 hours followed by a 4-hour treatment with a DR5-agonistic antibody leads to significantly more cells with subG_1_ DNA content then cells treated with vehicle, ONC201 alone, or DR5-agonistic antibody alone (Figure 7B, Supplementary Figure 9C). A DR4-agonist antibody did not synergize with ONC201 in these experiments (Supplementary Figure 10). These results indicate that alternative TRAs such as a DR5-agonistic antibody can also be used to convert the response of breast cancer cells to ONC201 from anti-proliferative to pro-apoptotic.

## DISCUSSION

ONC201 is a promising new anticancer therapy currently being clinically tested in a variety of tumor types, including those of the breast. ONC201 is capable of dually inducing the TRAIL pathway through upregulation of the TRAIL ligand [1] and its surface receptor DR5 [11]. Breast cancers are known to be resistant to TRAIL [12] and the effects of ONC201 in the majority of breast cancers are anti-proliferative but not apoptotic. Our previous studies showed that *in vivo*, the apoptotic effect of ONC201 leads to efficacy of the compound while the anti-proliferative effect does not [18]. These observations suggested to us that ONC201 may show decreased efficacy as a single agent in breast cancers where the effects of the compound are anti-proliferative but not apoptotic. The goal of the present study was to identify strategies for converting the anti-proliferative effect of ONC201 in breast cancer into an apoptotic one. Such a strategy has the potential to induce durable regressions in patients whose tumors do not undergo apoptosis with ONC201 alone. Our approach was to profile the effects of ONC201 on the TRAIL pathway in TRAIL-resistant non-TNBC cells, looking for a block in pathway activation that could be targeted and reversed.

Our data show that the effects of ONC201 in TRAIL-resistant non-TNBC cells are anti-proliferative, involving a decrease in cell viability, a decrease in the incorporation of BrdU into newly synthesized DNA, and a G_1_ arrest in the absence of apoptosis. This phenotype is in agreement with our previous descriptions of the anti-proliferative effect of the compound [11, 18]. A recent paper focused on elucidating the mechanism of ONC201 in breast cancer found that the compound inhibited cell viability without inducing apoptosis in both TNBC and non-TNBC cells [26]. These findings are in agreement with our previous work in breast cancer [18] and the work presented here. Our *in vitro* studies did not extend beyond a 72-hour treatment with ONC201, as previous studies indicated that cells sensitive to the apoptotic effects of the compound died following a treatment of this duration [1, 11, 18, 27]. Interestingly, another group found that following 120 hours of *in vitro* treatment with the compound, breast cancer cells underwent a non-apoptotic death due to disrupted mitochondrial function [26]. While this is a novel finding, we are unsure that this mechanism is relevant *in vivo*, as our previous work in breast cancer showed that ONC201 inhibited the growth of xenografted tumors only if the effects of the compound *in vitro* involved caspase-8 cleavage and TRAIL-dependent apoptosis [18].

Our results show that ONC201 treatment of TRAIL-resistant non-TNBC cells leads to a small but significant upregulation of pro-death ligand TRAIL at the RNA and surface protein levels. Our data also show that TRAIL upregulation by ONC201 in breast cancer cells in which the effects of ONC201 are apoptotic is small, at less than 2-fold above vehicle. Non-TNBC cells are known to be resistant to TRAIL [12], and we previously showed that the cells used here are TRAIL resistant [18]. We reasoned that a resistance mechanism blocking signal transduction through the TRAIL pathway was responsible for our observation that despite TRAIL induction following ONC201 treatment, the effects of the compound were anti-proliferative but not apoptotic.

Unexpectedly, our data showed that ONC201 treated TRAIL-resistant non-TNBC cells became primed by the compound to undergo apoptosis through the extrinsic pathway. Low levels of surface DR5 are known to contribute to TRAIL-resistance in breast cancer [13] and we hypothesized that ONC201 treatment of TRAIL-resistant non-TNBC cells was not leading to upregulation of the receptor. This was not the case, as our data show that treatment of TRAIL-resistant non-TNBC cells potently upregulates DR5 at both the RNA and protein level. ONC201 treatment leads to DR5 upregulation through activation of an ISR. Previous work from our lab showed that upregulation of DR5 at the mRNA level following ONC201 treatment in colorectal cells was blocked by knockdown of ISR mediators ATF4 and CHOP [11] and that ONC201 treatment activates an ISR in breast cancer cells [18]. We can speculate that the mechanism by which ONC201 upregulates DR5 in TRAIL-resistant non-TNBC cells is through activation of the ISR.

Expression of high levels of anti-apoptotic proteins can contribute to TRAIL resistance. Interestingly, our data show that ONC201 treatment leads to a decrease in the expression of the anti-apoptotic proteins in TRAIL-resistant non-TNBC cells. Interestingly, previous work from our lab showed that ONC201 treatment led to a decrease in c-FLIP expression in colorectal cancer cells [28] and that IAP and Bcl-2 family proteins may play a role in tumor cell sensitivity to ONC201 [29]. cFLIP prevents proper formation of the death inducing signaling complex (DISC) by competing with caspase-8 for binding to FADD associated with death receptors [14], and it would be interesting to further explore the relevance of c-FLIP in the observed sensitization of breast cancer cells to rhTRAIL by ONC201. Additional work from our lab showed that in colorectal and lung cancer cells downregulation of XIAP following ONC201 treatment correlated with whether the effects of the compound were apoptotic or anti-proliferative. Despite this correlation, knockdown of XIAP did not convert the response of cancer cells to ONC201 from anti-proliferative to apoptotic and overexpression of XIAP did not convert the response from apoptotic to anti-proliferative, indicating that XIAP levels alone do not determine cellular response to ONC201 [11]. Our current data further strengthen this conclusion, as XIAP is downregulated by ONC201 in breast cancer cells even in the absence of apoptosis induction. Our data also show that changes in expression of anti-apoptotic RNA transcripts do not correlate with changes in anti-apoptotic protein levels. This observation suggests that protein levels are being downregulated posttranscriptionally or posttranslationally. In glioblastoma, downregulation of Mcl-1 by ONC201 was shown to be mediated posttranslationally [30]. Further studies will elucidate the mechanisms of action responsible for the observed effects of the compound on anti-apoptotic proteins in breast cancer.

Despite apoptotic priming, the effects of ONC201 in TRAIL-resistant non-TNBC cells remain anti-proliferative and not apoptotic. This led us to hypothesize that the relatively low amount of TRAIL induced following ONC201 treatment was insufficient to trigger cell death. The addition of exogenous rhTRAIL to ONC201 treated TRAIL-resistant non-TNBC cells led to a striking conversion of the response of the cells to the compound from anti-proliferative to apoptotic in a DR5-dependent manner. *In vivo*, the growth rate of tumors treated with a combination of ONC201 and rhTRAIL was slowed when compared with a vehicle control. A therapeutic strategy capable of changing the response of cancer cells to ONC201 from anti-proliferative to apoptotic is a novel approach with high clinical relevance. Our data show that the effect extends beyond breast cancer, as the response of colorectal, lung, pancreatic, and glioblastoma cells to ONC201 can also be converted from anti-proliferative to apoptotic with the addition of rhTRAIL. The findings in glioblastoma are of particular interest, as ONC201 has shown preliminary efficacy in patients with recurrent H3 K27M-mutant high-grade glioma [31–33]. Biomarker studies showed that apoptosis was only induced in a subset of glioma tumors from patients treated with ONC201 [32], suggesting the need for a combinatorial therapeutic strategy. Importantly, the addition of rhTRAIL to an ONC201-treated normal fibroblast cell line does not convert the response of the cells to ONC201 from anti-proliferative to apoptotic. This finding suggests that the combination will be non-toxic to normal cells. *In vitro,* there have been concerns regarding the hepatotoxicity of rhTRAIL [34]. However, we did not observe changes in the liver architecture or morphology that would indicate hepatotoxicity in mice treated with three cycles of 100 mg/kg ONC201, 5 mg/kg rhTRAIL, or the combination (Supplementary Figure 6B). Although preliminary biomarker studies in MDA-MB-361 xenografts treated once with ONC201, rhTRAIL, and the combination did not show decreased Ki67 staining intensity or decreased cleaved caspase-3 positive cell count (data not shown), the mice had received only one cycle of treatment. Studies in the MDA-MB-436 and MDA-MB-231 breast cancer models did show decreased Ki67 staining intensity in tumors from mice treated with the combination of ONC201 and rhTRAIL when compared with a vehicle control.

Our data show that ONC201 treatment leads to the recruitment of NK cells to breast cancer tumors *in vivo*. These observations complement previous work from our lab, which showed that ONC201 treatment led to the recruitment of NK cells to colorectal tumors in mice [20], as well as data from a clinical of ONC201 as a single agent which showed further immunostimulatory effects of the compound [8]. We also showed that NK cells exhibit a greater cytotoxicity against ONC201-treated breast cancer cells when compared with vehicle treated cells. Further studies to elucidate the mechanism behind this observed increase in cytotoxicity will strengthen our findings.

rhTRAIL is no longer being tested clinically. For this reason, we wanted to test the ability of other TRAIL receptor agonists (TRAs) to convert the response to ONC201 from anti-proliferative to apoptotic. The DR5-agonistic antibody tigatuzumab was recently tested in combination with nanoparticle albumin-bound paclitaxel in patients with TNBC [35]. Our data show that anti-DR5 antibody lexatumumab (but not anti-DR4 antibody mapatumumab) is highly effective at converting the response of TRAIL- or lexatumumab-resistant non-TNBC cells to ONC201 from anti-proliferative to apoptotic. These results suggest that alternate TRAs may be used to convert the response of ONC201 from anti-proliferative to apoptotic. Future studies testing the combination of ONC201 and clinically relevant TRAs *in vitro* and *in vivo* will pave the way for the clinical translation of such a combination.

Our previous data showed that in breast cancer, the anti-proliferative effects of ONC201 are more common than the apoptotic effects. While the apoptotic effects of ONC201 led to *in vivo* efficacy of the compound, the anti-proliferative effects did not [18]. In our present study, we investigated mechanisms as well as strategies to convert the response of breast cancer cells to ONC201 from anti-proliferative to pro-apoptotic. Here we show that TRAIL-resistant non-TNBC cells are primed by ONC201 to undergo apoptosis through the extrinsic pathway, expressing high levels of DR5 and low levels of anti-apoptotic proteins. TRAIL receptor agonists such as rhTRAIL or a DR5-agonistic antibody convert the response of these cells to ONC201 from anti-proliferative to apoptotic. These findings may have clinical relevance as ONC201 is currently being tested in patients with breast cancer, and we believe that this newly identified combinatorial strategy has the potential to induce tumor regressions in patients with limited response to ONC201 monotherapy.

## MATERIALS AND METHODS

### Cell culture and reagents

Cancer cell lines used in this study were obtained from the Fox Chase Cancer Center cell culture facility or directly from the American Type Culture Collection. The E0771 mouse breast tumor line specifically was obtained from CH3 BioSystems, and the NK92 cell line was provided by the Kerry Campbell laboratory. The cell lines were authenticated using short tandem repeat profiling (IDEXX BioResearch) and were confirmed to be mycoplasma free. ONC201 was supplied by Oncoceutics, Inc., and was dissolved in DMSO. DR5-agonistic antibody lexatumumab was supplied by Human Genome Sciences and was dissolved in PBS. rhTRAIL was generated as described [36].

### Quantitative reverse transcription-polymerase chain reaction (qRT-PCR)

To determine cellular DR5 and TRAIL mRNA expression, total RNA was isolated from cells using the RNeasy mini kit (QIAGEN) according to the manufacturer’s protocol. The extracted RNA was quantified using a NanoDrop spectrophotometer (Thermo Fischer Scientific). RNA was converted to cDNA using the PrimeScript 1st strand cDNA synthesis kit (Clontech) according to the manufacturer’s protocol. Amplified cDNA was added to a 384 well plate along with oligonucleotide primer and SYBR green PCR master mix (Applied Biosystems). The C_t_ values were calculated using a 7900HT Fast Real-Time PCR system (Thermo Fischer Scientific). The fold change in gene expression was calculated using the 2^–ΔΔCt^ method. The sequence of the oligonucleotide primers are: GAPDH forward: 5′-CTGGGCTACACTGAGCACC-3′, GADPH reverse: 5′-AAGTGGTCGTTGAGGGCAATG-3′. DR5 forward: 5′-ACAGTTGCAGCCGTAGTCTTG-3′, DR5 reverse: 5′-CCAGGTCGTTGTGAGCTTCT 3′.

### Flow cytometric analysis of cell surface protein levels

Surface staining was performed as described [18].

### Cellular immunofluorescence

To visualize cellular caspase-3 cleavage, cells were plated in chamber slides and allowed to adhere overnight prior to treatment. Following treatment, the cells were fixed in a 1:1 solution of methanol and acetone. Fixed cells were blocked and then stained with a primary antibody from Cell Signaling Technologies (rabbit anti-cleaved caspase-3, cat # 9661) overnight at 4°C. After washing, a secondary antibody from Invitrogen (Alexa Fluor 568 goat anti-rabbit IgG, cat # 11077) was added to the cells for 2 hours at room temperature in the dark. Nuclei were stained with DAPI and ProLong Gold Antifade Mountant (Thermo Fischer Scientific) used as a mounting medium. The slides were imaged using a Leica TCS SP8 confocal laser scanning microscope (Leica Microsystems).

### Western blotting

As described by Ralff *et al*. [18], cells were harvested and lysed. Protein was quantified and then samples were prepared for gel loading. Samples were loaded into and run on 4–12% SDS-polyacrylamide gels. Proteins were then transferred to polyvinylidene difluoride membranes. Membranes were incubated overnight with primary antibodies, then with horseradish peroxidase–labeled secondary antibodies. Syngene imaging system was used to detect chemiluminescent signal. Antibodies used were as follows: Bcl-xL, CST #2764; c-IAP1, CST #7065; FLIP, CST #8510; Mcl-1, CST #5453; XIAP, cst #14334; survivin, CST #2808; beta-actin, Abcam #8229; PARP, CST #9532; caspase-8, CST #9746; cleaved caspase-8, CST #9496; caspase-9, CST #9502; caspase-3, CST #9665; cleaved caspase-3, CST #9661; DR5: CST #3696.

### Flow cytometric BrdU-PI and PI staining

To assess the effects of ONC201 on cellular proliferation and cell cycle profile, pre-treated cells were pulsed with a final concentration of 10 μM BrdU in complete culture media for 30 minutes at 37°C. Staining was performed as previously described [18]. 20,000 cells were counted. FlowJo software was used to exclude doublets and perform data analysis. To quantitate the subG_1_ population of cells and thus indicate apoptosis, propidium iodide (PI) staining and flow cytometric analysis was used. Cells were fixed in 70% ethanol and stained with propidium iodide in the presence of ribonuclease A (RNase A). The cells were analyzed using an Elite Epics flow cytometer (Beckman Coulter). Cell cycle analysis was performed using FlowJo software.

### siRNA-mediated DR5 knockdown

To determine the importance of DR5 upregulation by ONC201 in the ability of rhTRAIL to convert response to the compound from anti-proliferative to apoptotic, a reverse transfection protocol was used to transfect scrambled or DR5 targeting siRNA (Santa Cruz Biotechnology, scrambled cat # 37007, DR5 targeting cat # 40237) into cells. Lipofectamine-3000 (Invitrogen) was incubated with siRNA according to the manufacturer’s protocol to form siRNA-lipid complexes. siRNA-lipid complexes were added to cells suspended in antibiotic free medium. The cells were incubated with the complexes for 18 hours then were treated with a vehicle control or ONC201 in complete medium.

### Cell viability assays

Cell viability assays were performed as described [18]. For the combination experiments, after 72 hours the media was removed from the ONC201 treated wells and replaced with fresh media containing a vehicle control or one of three doses of rhTRAIL. 4 hours later, cellular viability was quantitated using the CellTiter-Glo luminescent cell viability assay (Promega) according to the manufacturer’s instructions. GraphPad Prism software was used to generate dose-response curves.

### Tumor model studies

To investigate whether the combination of ONC201 and rhTRAIL showed efficacy and whether ONC201 treatment led to recruitment of NK cells to tumors, animal studies were performed. The methods outlined by Ralff *et al.* [18] were utilized for the MDA-MB-436, MDA-MB-231, MDA-MB-361 xenograft models. In brief, 6–8-week-old female athymic nude mice from Taconic (NCrFoxn1^nu^, genotype sp/sp) had their mammary fat pads inoculated with breast cancer cells. The mice were randomized and treatment was initiated once the tumors reached a volume of 150 to 250 mm^3^. For the syngeneic E0771 model, C57BL/6 mice were used in place of athymic nude mice. Tumor volumes were measured with calipers as (w^2^ × l)/2 where w is tumor width and l is tumor length. Between 5 and 7 tumors were included per group.

### Immunohistochemistry

#### Staining

Tumors were harvested 24 hours after treatment with rhTRAIL. They were fixed in formalin immediately after harvesting in cassettes. After fixation, cassettes were paraffin embedded. Slides were cut 5 μm thick. IHC was initiated by deparaffinizing slides using xylene. Slides were dehydrated through sequential dilutions of ethanol. The antigen retrieval step was conducted by heating slides for 10 minutes in pH 6.0 citrate acid buffer. Cleaved caspase-3 antibodies were obtained from BD Biosciences, used at 1:100 dilution. Ki67 (MIB-1) antibody was obtained from Cell Signaling Technologies, used at 1:200 dilution. Slides were incubated in primary antibodies overnight; respective secondary antibodies were added the following day. Slides were developed using DAB Staining Kit (Vector Labs) and mounted using a xylene-based mount, Cytoseal XYL.

#### Cleaved caspase-3 quantification

Images were opened in ImageJ and stacked to RGB (Image > Color > Stack to RGB). After image stacking, the colors were deconvolved using ImageJ’s in-built color deconvolution option. All slides had been developed using DAB and counterstained using H&E. Image colors were separated using the H&E DAB option. After the color separation, only the DAB image was used, and threshold for particle detection was set at 100. All particles less than 20 px^2^ were excluded to prevent any debris from being counted. Apoptotic cells were then automatically counted by ImageJ. Each image was visually confirmed for proper counting and analyses.

#### Ki-67 quantification

Slides were quantified using QuPath, an open-source, automated program for IHC quantification [37]. For binary Ki67 staining, intensity threshold was set to Nuclear DAB optical density (OD) Mean of 0.2. For intensity threshold of ‘low,’ ‘medium,’ and ‘high’ Nuclear DAB OD Mean were set to 0.2, 0.4, and 0.6, respectively. QuPath’s nuclear segmentation algorithm was used and visual confirmation was done on every slide.

### Flow cytometric analysis of tumor infiltrating lymphocytes

Three weeks after the initiation of treatment with a vehicle control or ONC201, the mice bearing E0771 tumors were sacrificed. Their tumors were removed and were crushed and filtered. Dissociated cells were collected and incubated with red blood cell lysis buffer. Afterwards, cells were washed with FACS buffer and incubated for 10 minutes on ice with a mouse CD16/CD32 specific antibody to block Fc receptors (eBioscience, cat # 14-0161-81). Cells were then incubated with primary antibodies from eBioscience for 30 minutes on ice in the dark (CD45: cat # 63-0451-82, CD19: cat # 48-0193-82, CD3: cat # 47-0032-82, NK1.1: cat # 17-5941-82). Cells were washed and resuspended in FACS buffer with 1 μg/mL propidium iodide added for dead cell exclusion. The cells were analyzed using an LSR II flow cytometer (BD Biosciences), and FlowJo software was used to perform data analysis.

### Cell-mediated cytotoxicity assay

To determine the effects of ONC201 treatment on killing of breast cancer cells by NK cells, T47D cells were pre-treated for 48 hours with a vehicle control or 10 μM of ONC201. A LIVE/DEAD cell-mediated cytotoxicity kit (Life Technologies) was used according to the manufacturer’s protocol to determine NK92 cytotoxicity against the pre-treated cancer cells.

### Statistical analysis

For the *in vivo* growth rate analysis, tumor volume (V) measured over time (*t*) was assumed to follow the exponential model, V (*t*)=*V*_0_ × *exp* (*t* × β), where *V*_0_ is the volume at time of treatment initiation and β is the growth slope parameter. *V*_0_ and β were estimated separately for each mouse by least squares. Two sample *t*-tests were used to statistically compare mean growth slopes (β’s) between pairs of treatment conditions, separately by experiment. In secondary analyses we combined the data from the two experiments and used general linear models to conduct pairwise treatment group comparisons of growth rates. Covariates included treatment group and experiment number to account for variability in growth rates across experiments. All tests were two-sided with a 5% type I error. Analyses were conducted using R and SAS version 9.4. To assess the statistical significance of all other differences, Prism 7 software (GraphPad) was used to perform unpaired *t* tests. Comparisons were made against the vehicle-treated control.

## SUPPLEMENTARY MATERIALS



## Notes

**Editorial note**

This paper has been accepted based in part on peer-review conducted by another journal and the authors' response and revisions as well as expedited peer-review in Oncotarget.
