# Supplementary material for: TRAIL receptor agonists convert the response of breast cancer cells to ONC201 from anti-proliferative to apoptotic
Source: Oncotarget. 2020 Oct 20;11(42):3753–69. doi: 10.18632/oncotarget.27773 (PMC7584235; doi:10.18632/oncotarget.27773)
Supplement: Supplementary file 1 [file oncotarget-11-3753-s001.pdf]

TRAIL receptor agonists convert the response of breast cancer cells to ONC201 from anti-proliferative to apoptotic

SUPPLEMENTARY MATERIALS

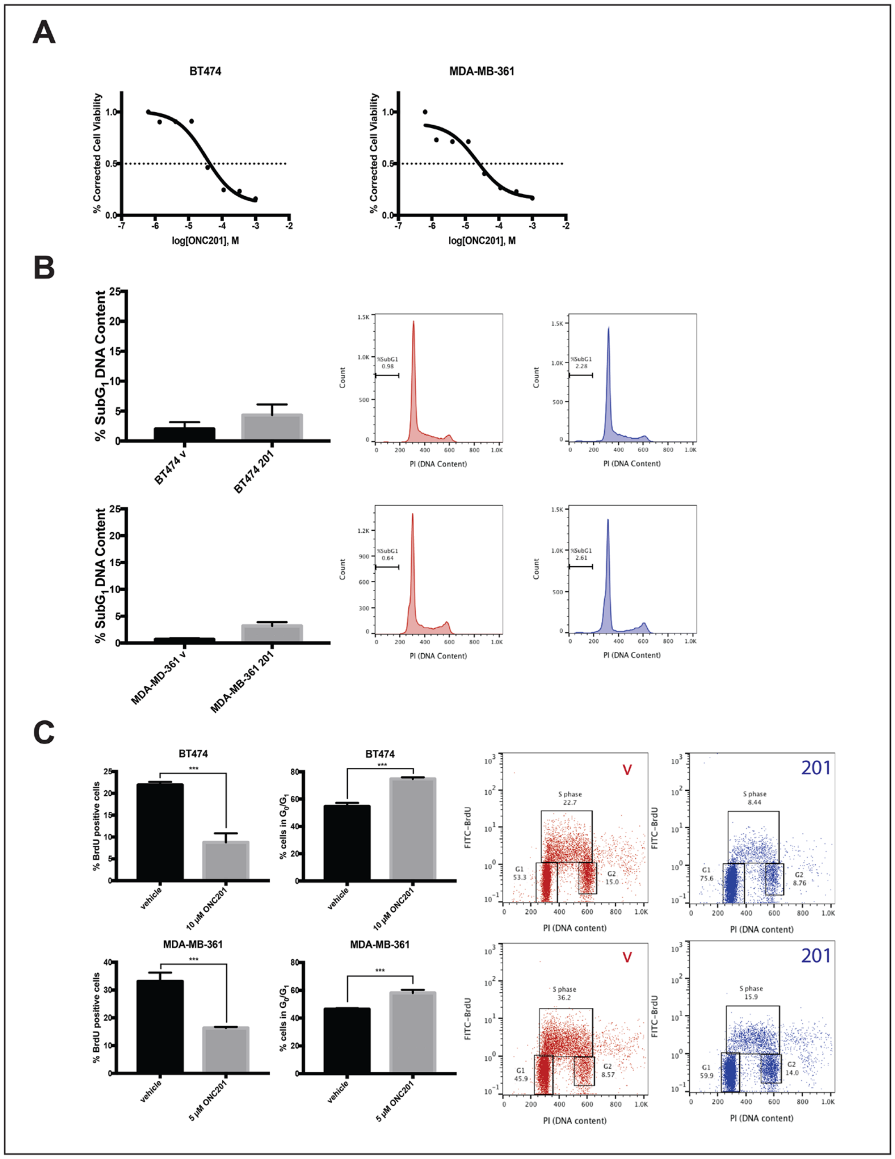

Supplementary Figure 1: ONC201 inhibits the proliferation but does not induce apoptosis in BT474 and MDA-MB-361 TRAIL-resistant non-TNBC cells. (**A**) Dose response curves for cells treated with varying concentrations of ONC201 for 72 hours were generated. Cell viability was determined using CellTiterGlo reagent. (**B**) Cells were treated with a vehicle control or approximate GI_{50} doses of ONC201 for 72 hours and stained with propidium iodide. Flow cytometric analysis of the cells was used to determine the percentage of cells with subG_1 DNA content. (**C**) Cells were treated with a vehicle control or approximate GI_{50} doses of ONC201 for 48 hours, then pulsed with BrdU for 30 minutes. BrdU-PI staining was performed and the % BrdU positive cells quantitated using flow cytometric analysis. Representative dot plots are shown. ns: $p > 0.05$; $^{*}p \leq 0.05$, $^{**}p \leq 0.01$, $^{***}p \leq 0.001$, $^{****}p \leq 0.0001$.

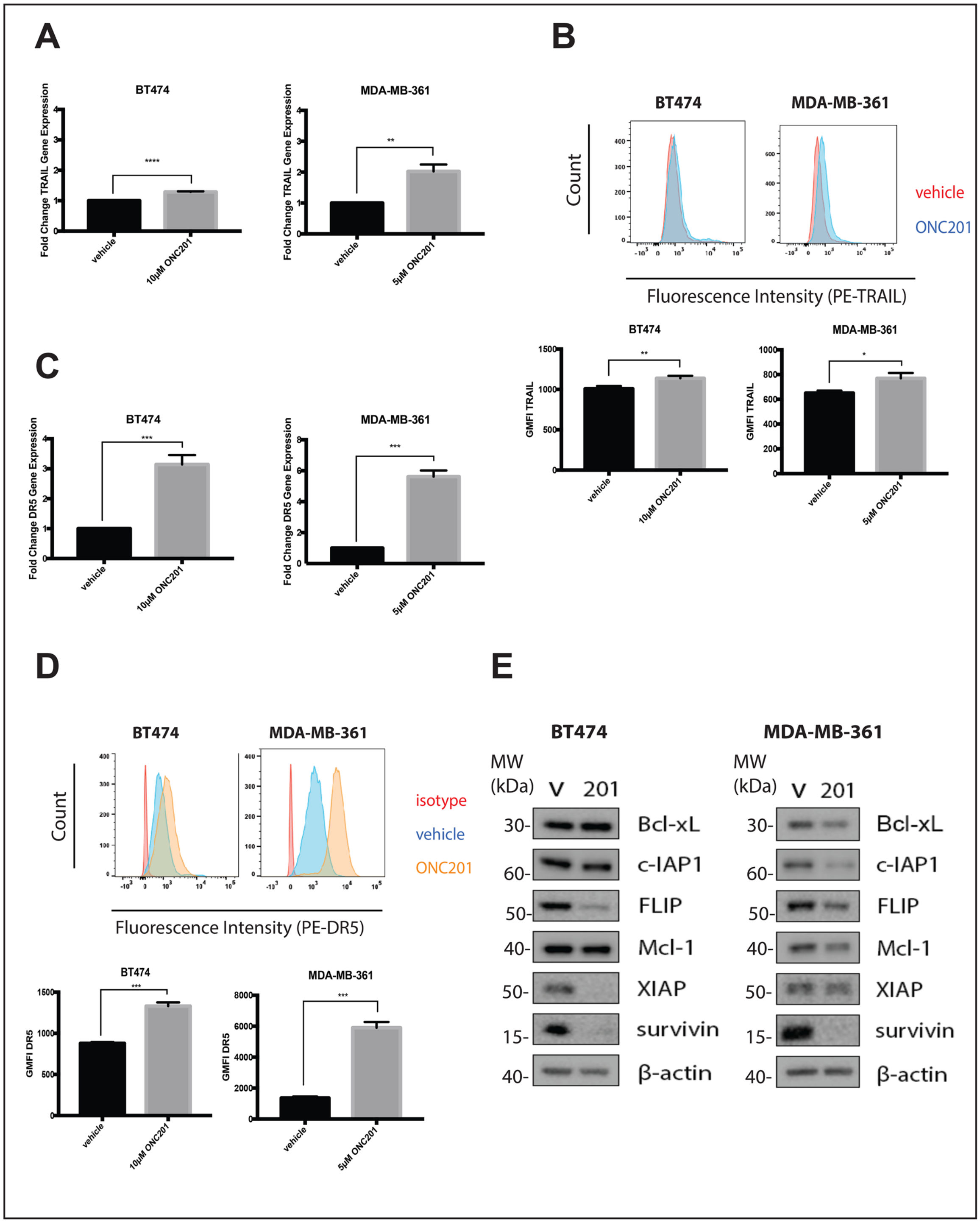

Supplementary Figure 2: ONC201 treated BT474 and MDA-MB-361 non-TNBC cells increase TRAIL expression and are primed to undergo TRAIL-dependent apoptosis. (**A**) Cells were treated with a vehicle control or approximate GI_{50} doses of ONC201 for 48 hours and qRT-PCR was used to determine the fold change in TRAIL gene expression. (**B**) Cells were treated with a vehicle control or approximate GI_{50} doses of ONC201 for 72 hours and then stained with an anti-TRAIL antibody. The geometric mean fluorescence intensity was determined using flow cytometric analysis. Representative histograms show the staining. (**C**) Cells were treated with a vehicle control or approximate GI_{50} doses of ONC201 for 48 hours and qRT-PCR was used to determine the fold change in DR5 gene expression. (**D**) Cells were treated with a vehicle control or approximate GI_{50} doses of ONC201 for 72 hours and then stained with an isotype control or an anti-DR5 antibody. The geometric mean fluorescence intensity was determined using flow cytometric analysis. Representative histograms show the staining. (**E**) Cells were treated with a vehicle control or GI_{50} doses of ONC201 for 72 hours. Western blot analysis used to assess expression of anti-apoptotic proteins. ns: $p > 0.05$; $^{*}p \leq 0.05$, $^{**}p \leq 0.01$, $^{***}p \leq 0.001$, $^{****}p \leq 0.0001$.

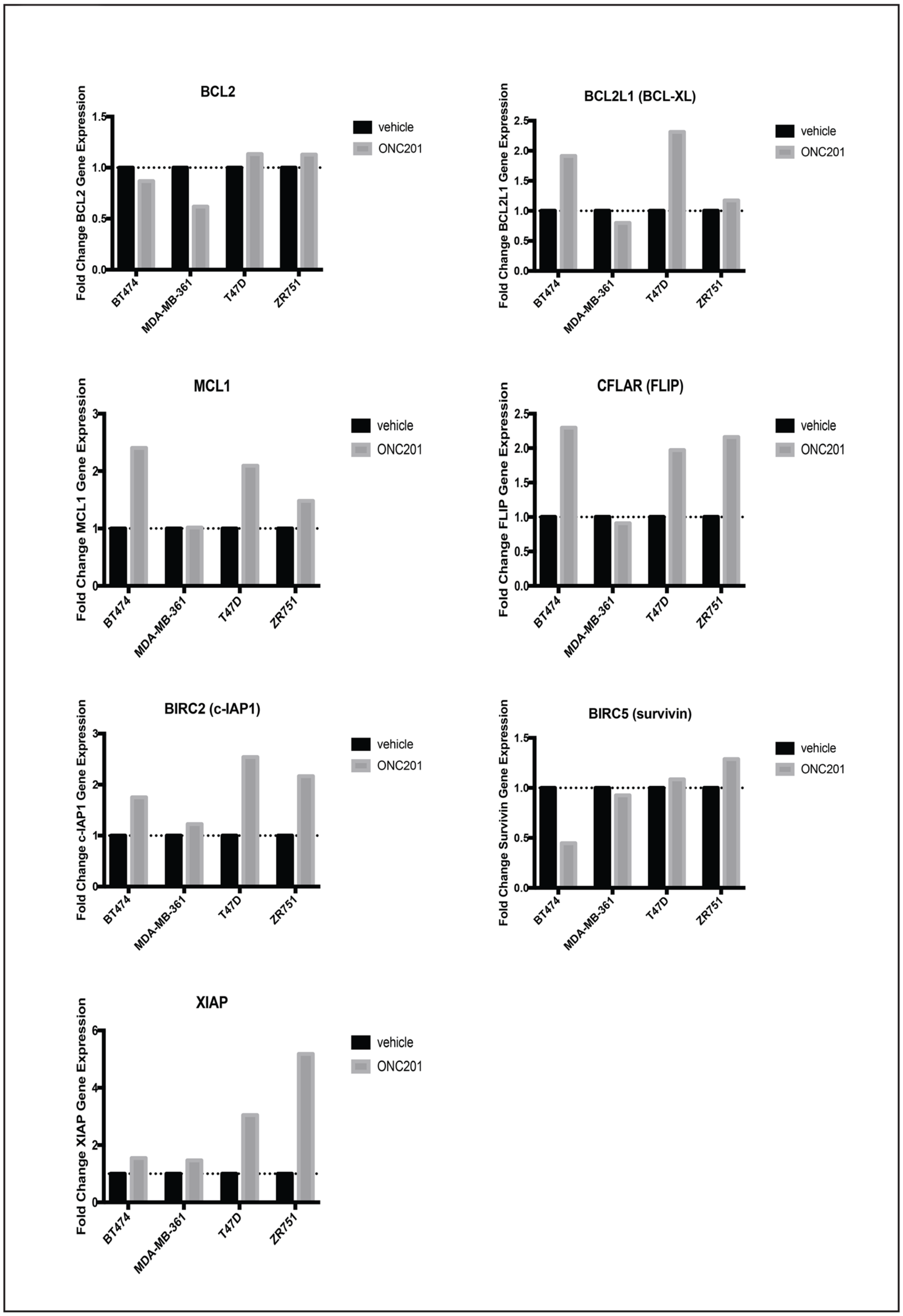

Supplementary Figure 3: The effects of ONC201 treatment on anti-apoptotic gene expression do not correlate with the compound's effects on protein levels. Cells were treated with a vehicle control or approximate GI_{50} doses of ONC201 for 48 hours and qRT-PCR was used to determine the fold change in anti-apoptotic gene expression.

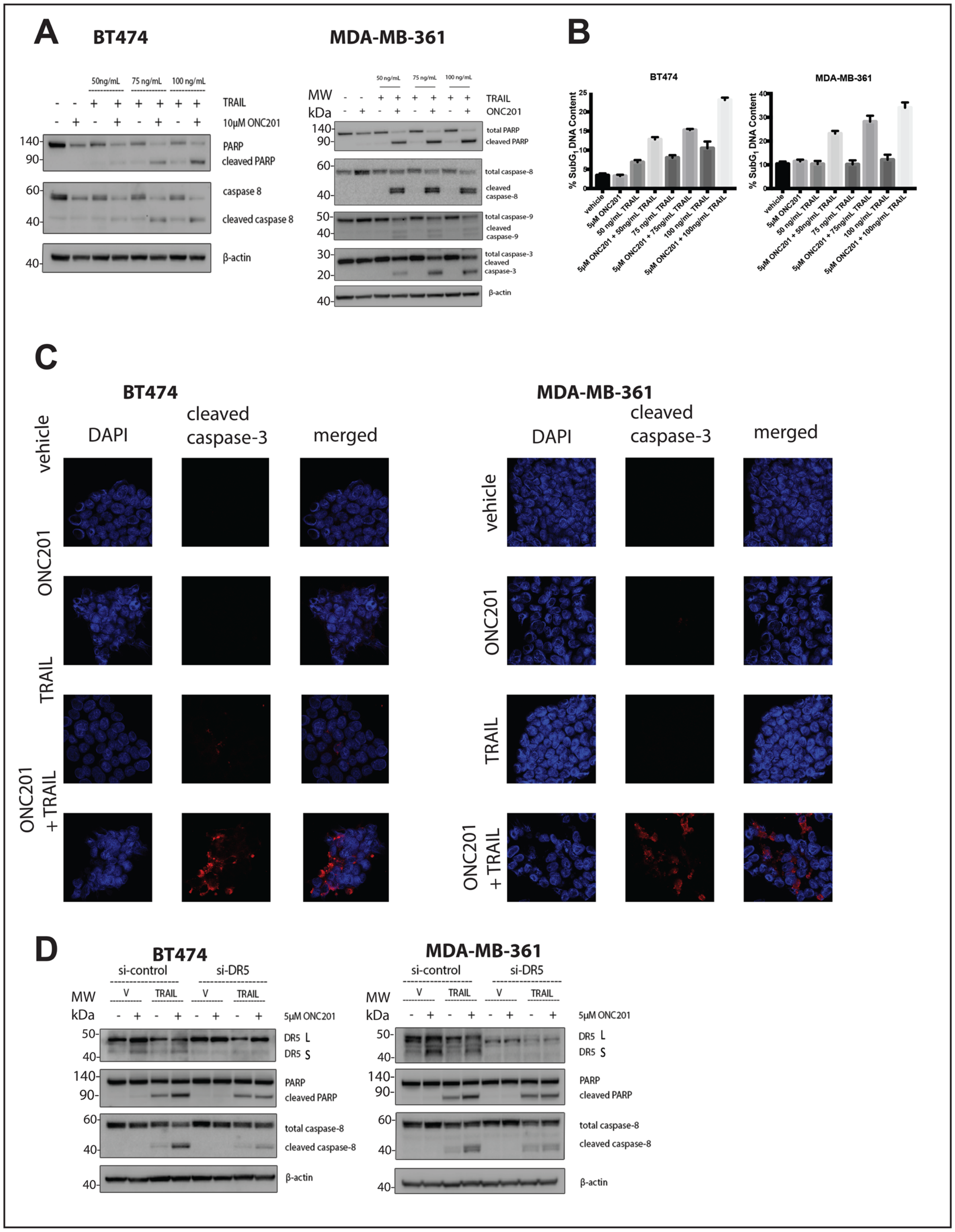

Supplementary Figure 4: Addition of rhTRAIL converts the response of BT474 and MDA-MB-361 breast cancer cells to ONC201 from anti-proliferative to pro-apoptotic in a DR5-dependent manner. (**A**) BT474 and MDA-MB-361 cells were treated with a vehicle control or GI_{50} doses of ONC201 for 72 hours. Recombinant human TRAIL (rhTRAIL) was added for 4 hours and the induction of cell death was assessed using western blot. (**B**) BT474 and MDA-MB-361 cells were treated as above and stained with propidium iodide. Flow cytometric analysis of the cells was used to determine the percentage of cells with $subG_1$ DNA content. (**C**) BT474 and MDA-MB-361 cells were transfected with a scrambled siRNA or a DR5-targeting siRNA for 18 hours, then treated with a vehicle control or GI_{50} doses of ONC201 for 48 hours. (**D**) Recombinant human TRAIL (rhTRAIL) was added for 4 hours and western blot analysis used to assess induction of cell death.

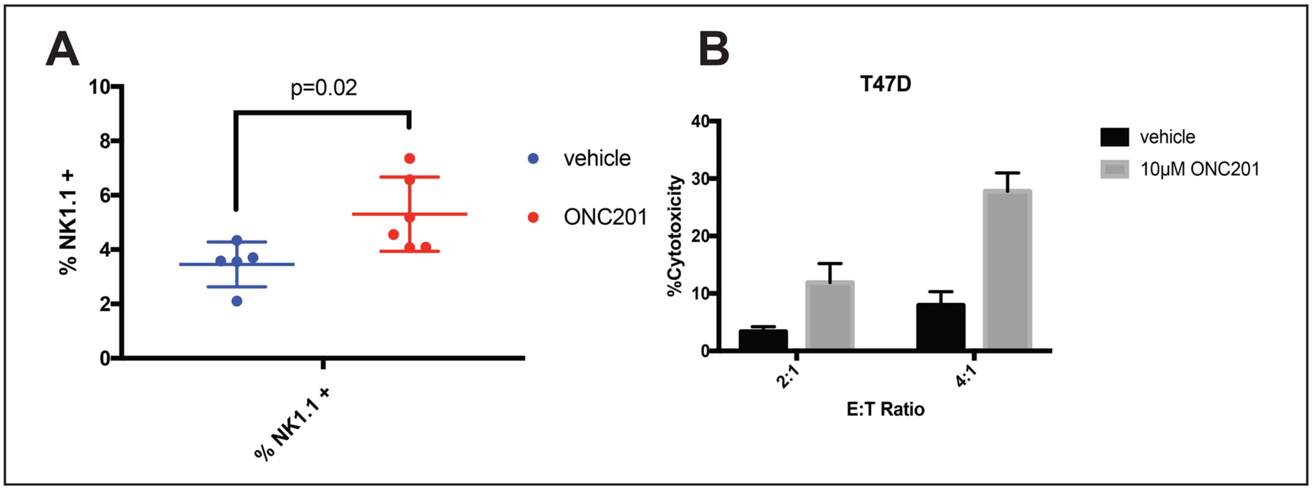

Supplementary Figure 5: NK cells exhibit greater cytotoxicity against ONC201-treated cells compared with vehicle treated cells. (**A**) Flow cytometric analysis of tumor infiltrating lymphocytes (TILs) from mice treated with a vehicle control or 100 mg/kg ONC201 orally twice weekly for 3 weeks. The percent of CD19-CD3-NK1.1+ lymphocytes are graphed. (**B**) Cell based cytotoxicity assay was used to quantify killing of tumor cells by NK92 cells. ns: $p > 0.05$; $^{*}p \leq 0.05$, $^{**}p \leq 0.01$, $^{***}p \leq 0.001$, $^{****}p \leq 0.0001$.

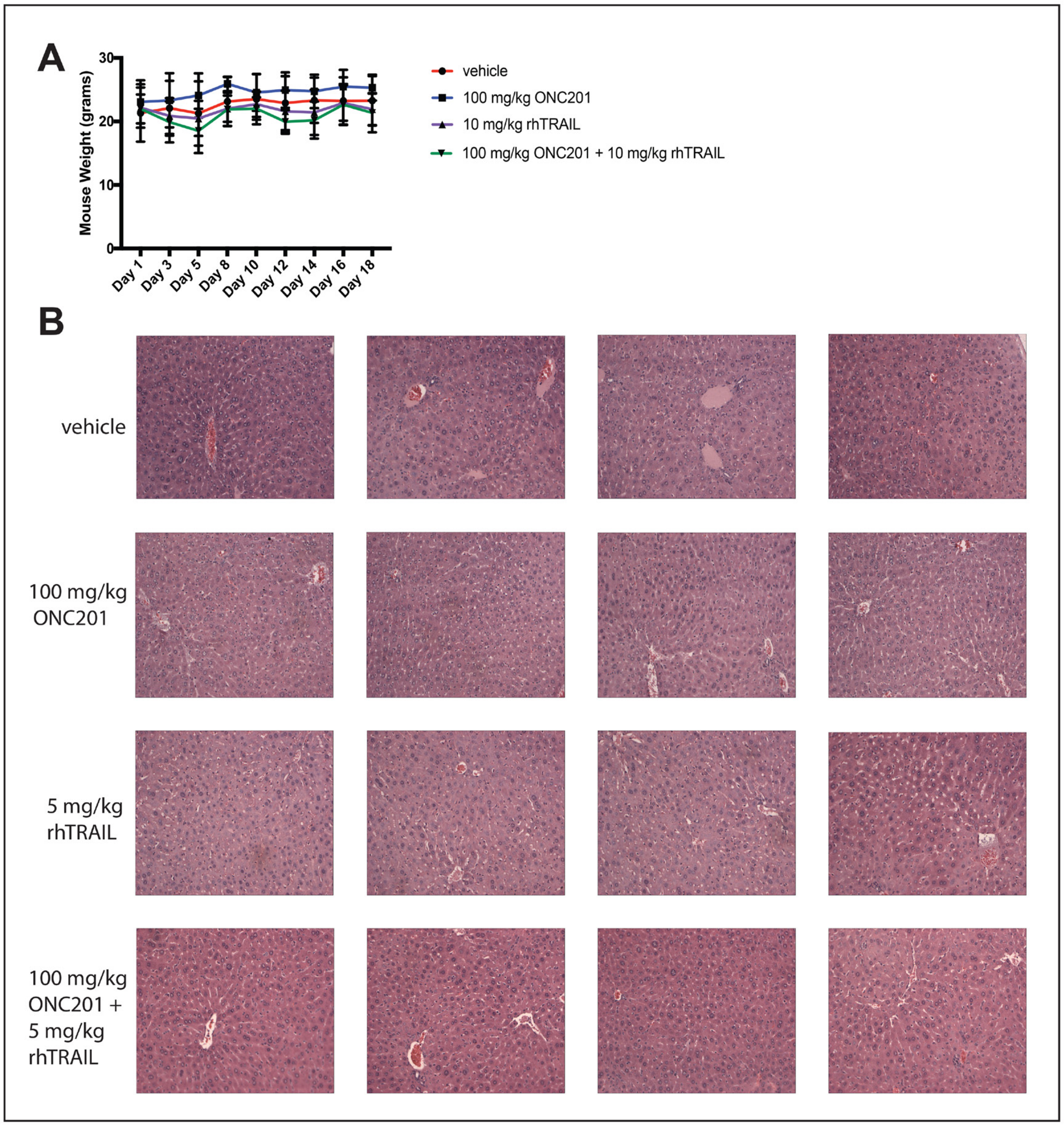

Supplementary Figure 6: Safety of ONC201 and rhTRAIL *in vivo*. (**A**) Mice bearing MDA-MB-361 xenograft tumors were treated weekly with a vehicle control, 100 mg/kg ONC201 orally, 10 mg/kg rhTRAIL intravenously, or a combination of ONC201 and rhTRAIL. rhTRAIL was given 72 hours after ONC201. Mouse weights were measured over time. (**B**) Liver sections from mice bearing MDA-MB-361 xenograft tumors treated with a vehicle control, 100 mg/kg ONC201 orally, 5 mg/kg rhTRAIL intravenously, or a combination of ONC201 and rhTRAIL for three cycles were stained with H&E 24 hours after treatment.

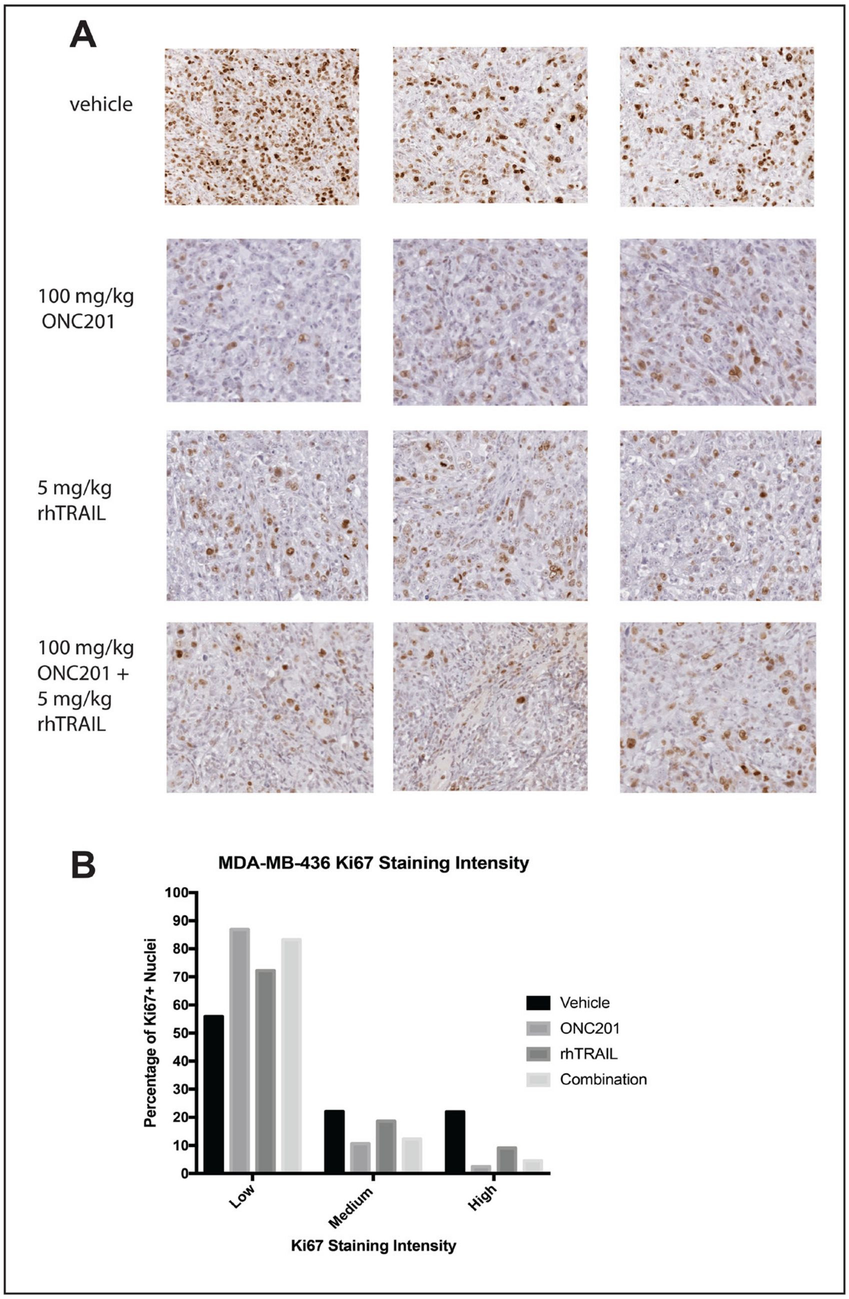

Supplementary Figure 7: Ki67 staining in MDA-MB-436. (**A**) Mice bearing MDA-MB-436 xenograft tumors were treated as treated with a vehicle control, 100 mg/kg ONC201 orally, 5 mg/kg rhTRAIL intravenously, or a combination of ONC201 and rhTRAIL for three cycles. 7 days after final TRAIL dose tumor sections were fixed and stained for Ki67. (**B**) Percentage of Ki67 positive nuclei staining with a low, medium, or high intensity MDA-MB-436 xenograft tumors was determined.

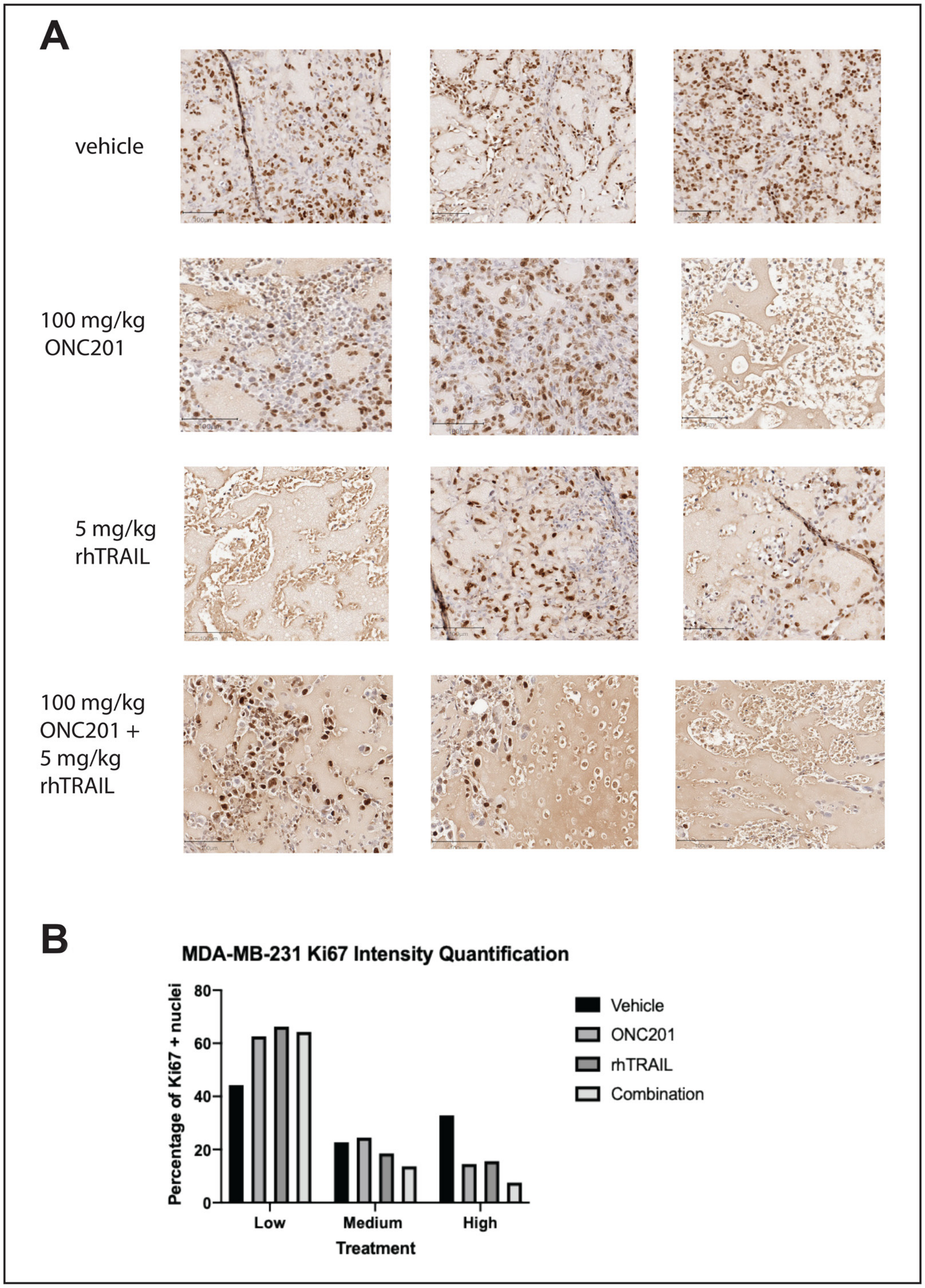

Supplementary Figure 8: Ki67 staining in MDA-MB-231. (**A**) Mice bearing MDA-MB-231 xenograft tumors were treated with a vehicle control, 100 mg/kg ONC201 orally, 5 mg/kg rhTRAIL intravenously, or a combination of ONC201 and rhTRAIL for one cycle. 24 hours after final TRAIL dose tumor sections were fixed and stained for Ki67. (**B**) Percentage of Ki67 positive nuclei staining with a low, medium, or high intensity MDA-MB-231 xenograft tumors was determined.

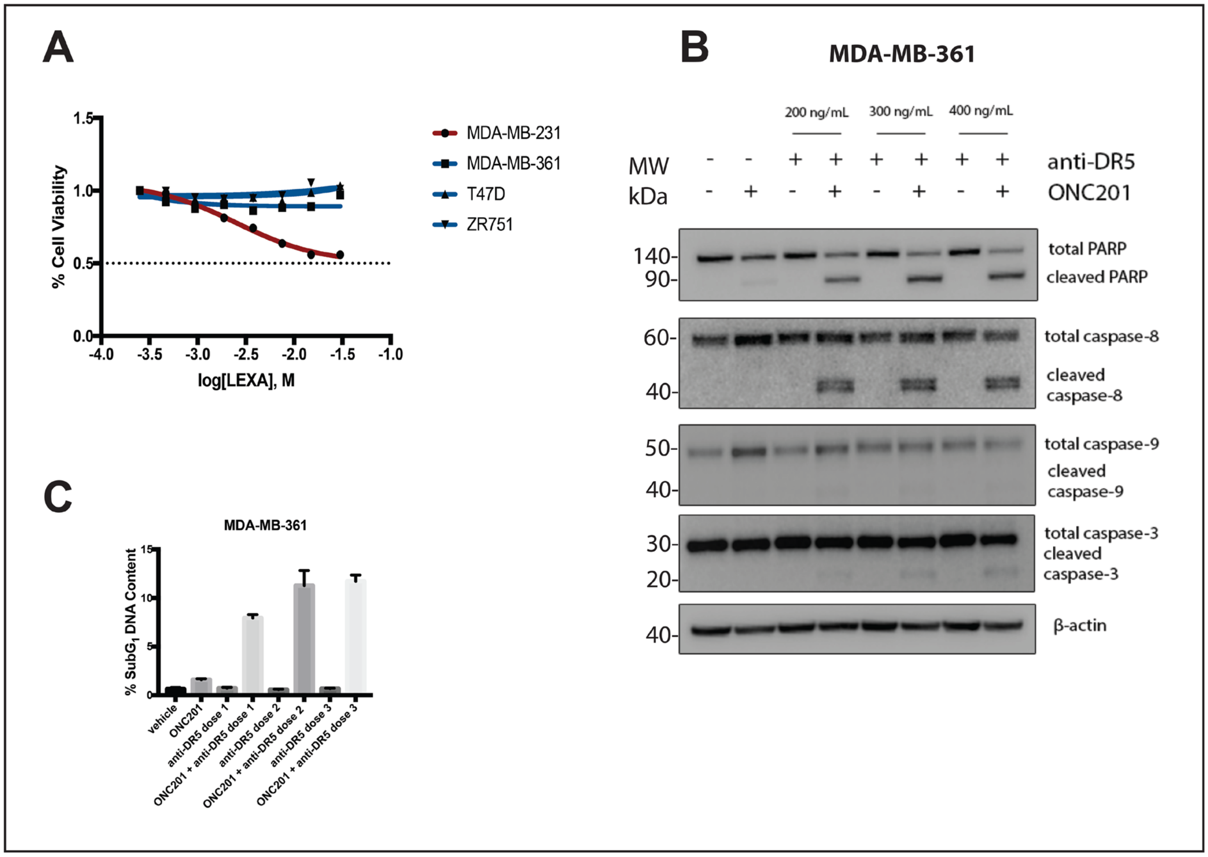

Supplementary Figure 9: A DR5-agonistic antibody can also be used to convert the response of other breast cancer cells from anti-proliferative to pro-apoptotic. (**A**) Breast cancer cells were treated with a vehicle control or increasing concentrations of anti-DR5 antibody lexatumumab for 72 hours. (**B**) MDA-MB-361 cells were treated as with a vehicle control or GI_{50} doses of ONC201 for 72 hours. Lexatumumab was added for 4 hours and the induction of cell death was assessed using western blot. (**C**) MDA-MB-361 cells were treated as above and stained with propidium iodide. Flow cytometric analysis of the cells was used to determine the percentage of cells with subG_1 DNA content.

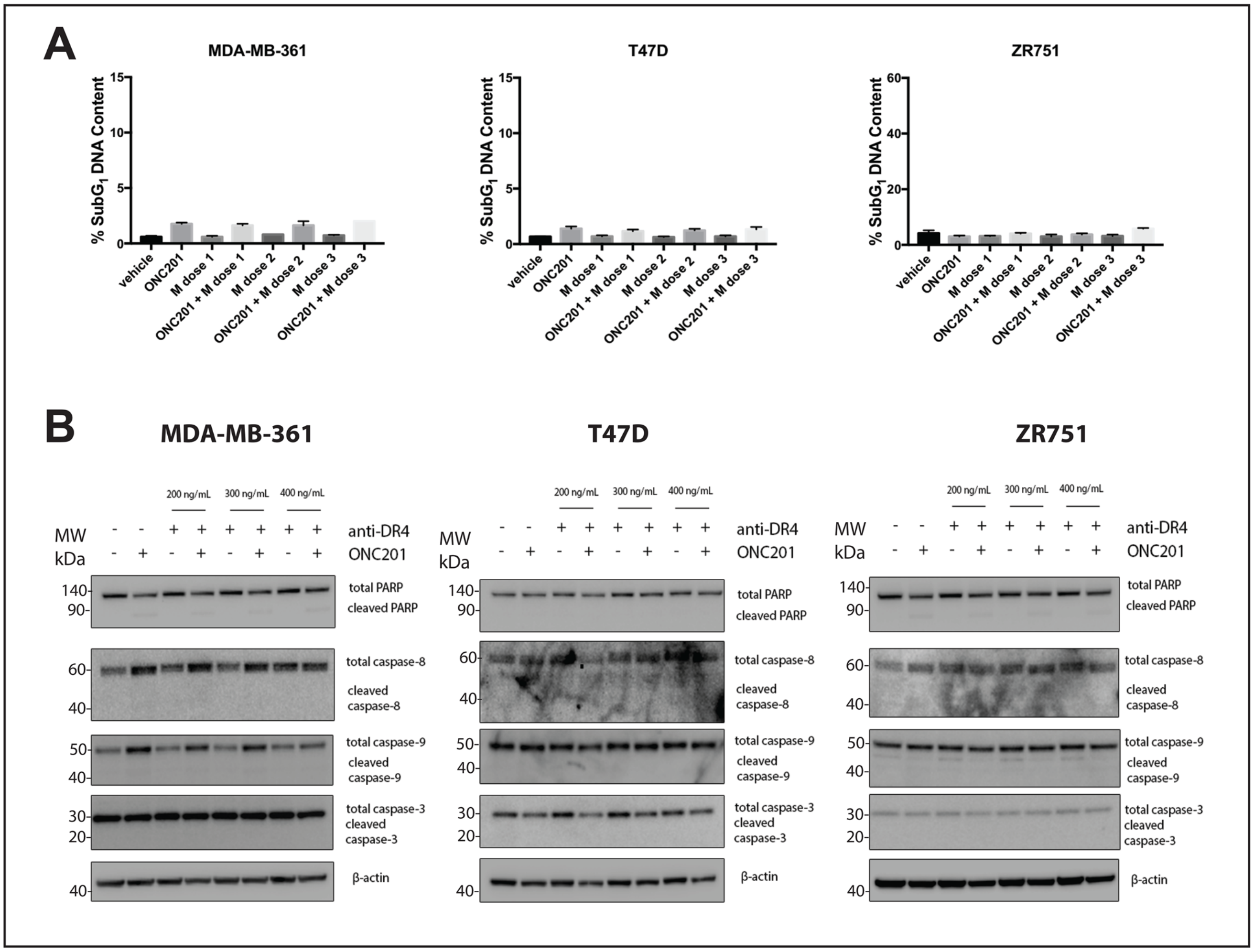

Supplementary Figure 10: A DR4-agonistic antibody does not convert the response of breast cancer cells from anti-proliferative to pro-apoptotic. (**A**) Breast cancer cells were treated with a vehicle control or increasing concentrations of anti-DR4 antibody mapatumumab for 72 hours and stained with propidium iodide. Flow cytometric analysis of the cells was used to determine the percentage of cells with subG_1 DNA content. (**B**) Breast cancer cells were treated as with a vehicle control or GI_{50} doses of ONC201 for 72 hours. Mapatumumab was added for 4 hours and the induction of cell death was assessed using western blot.
